# GLYDE-II: The GLYcan data exchange format

**DOI:** 10.1016/j.pisc.2016.05.013

**Published:** 2016-10-20

**Authors:** Rene Ranzinger, Krys J. Kochut, John A. Miller, Matthew Eavenson, Thomas Lütteke, William S. York

**Affiliations:** a Complex Carbohydrate Research Center, University of Georgia, USA; b Computer Science Department, University of Georgia, USA; c Institute of Veterinary Physiology and Biochemistry, Justus-Liebig-University Giessen, Germany

**Keywords:** Bioinformatics, Glycan, Structure, Representation, XML

## Abstract

The GLYcan Data Exchange (GLYDE) standard has been developed for the representation of the chemical structures of monosaccharides, glycans and glycoconjugates using a connection table formalism formatted in XML. This format allows structures, including those that do not exist in any database, to be unambiguously represented and shared by diverse computational tools. GLYDE implements a partonomy model based on human language along with rules that provide consistent structural representations, including a robust namespace for specifying monosaccharides. This approach facilitates the reuse of data processing software at the level of granularity that is most appropriate for extraction of the desired information. GLYDE-II has already been used as a key element of several glycoinformatics tools. The philosophical and technical underpinnings of GLYDE-II and recent implementation of its enhanced features are described.

## Introduction

Glycobiology, broadly defined as the study of the structure, biosynthesis and biological functions of glycans and glycoconjugates, is an emerging field of research that has found increasing applications in diverse technologies ranging from medicine to biofuels (Varki and Sharon, 2009; Walt et al., 2012). Glycomics, which focuses on the structures and abundances of specific glycans in various biological samples, has been enabled by developments in molecular analysis that make it possible to detect, identify and quantify glycans as free molecules or as components of glycoconjugates (Cummings and Pierce, 2014). However, the development of glycomics has lagged behind genomics and proteomics in large part due to analytical challenges that stem from the structural complexity of glycans. Glycan structures cannot be inferred directly from genomic data, as the structure of each glycan is the result of an often complex metabolic pathway whose individual steps are catalyzed by sundry glycosyl-transferases and other glycan modifying enzymes (Varki and Sharon, 2009).

Progress in glycomics has also been slowed by lack of robust informatics tools to archive, retrieve, analyze, mine and transfer the large amounts of multifaceted data that is generated in the course of this research (York et al., 2014). The development of databases and ontologies that contain knowledge regarding glycan structures and their relationships to biological and physical phenomena is especially challenging. Nevertheless, several isolated databases that archive information about carbohydrate structure, biosynthesis and function have been developed. The integration of this diverse information is a major bioinformatics challenge that must be addressed if glycobiology is to reach its potential to address critical issues in biomedicine, biofuels and other domains.

The existing carbohydrate databases implement diverse structural representation protocols. One reason for the development of different glycan sequence formats is the diversity of molecular building blocks (monosaccharides) and the frequent existence of branches. A second reason is that few of the sequence formats used in the various databases have been published. Unfortunately, this lack of accessibility has led to the proliferation of formats rather than to the establishment of a single standard. More than a dozen sequence formats have been developed, and more than one can be used in a single database or software application. These sequence formats include linearized representations of the branched sequences, such as LINUCS (Bohne-Lang et al., 2001), the BCSDB format (Egorova and Toukach, 2014) and LinearCode^®^ (Banin et al., 2002), as well as connection table representations, such as GlycoCT (Herget et al., 2008), WURCS (Tanaka et al., 2014) and KCF (Hashimoto et al., 2006) and XML representations, such as CabosML (Kikuchi et al., 2005). The development of GlycomeDB (Ranzinger et al., 2011), a meta-database for glycan structures, has been a major step towards the integration of this structural data. More recently, the GlyTouCan glycan structure repository (Aoki-Kinoshita et al., 2016) has been implemented to provide a robust and stable semantic basis for describing glycan structure. Nevertheless, communication between different data acquisition, storage and processing systems still requires well-defined methods for transferring structural information that may not be included in an existing database. The GLYcan Data Exchange format (GLYDE) has been developed as a standard XML (Extensible Markup Language) based format to address these concerns. GLYDE has been widely accepted by the glycoinformatics community (Packer et al., 2008) and is a frequently used format for exchanging glycan structure information (Eavenson et al., 2015). This paper describes the philosophical and technical background for GLYDE and the recently implemented enhancements of this standard.

## Results

The GLYDE XML format is defined via two schema specifications: an XSD (XML Schema Definition) schema and a complementary but more flexible DTD (Document Type Definition) schema. These define a specification framework, which we call PARCHMENT (PARtonomy of CHeMical ENTities), which allows the structure of biological molecules (including complex glycans) to be completely and unambiguously specified at several levels of granularity. Notably, this provides a *unified* format for the concise and complete structural representation of each molecule in the vast, naturally occurring combinatorial set of glycoconjugates that arises by the attachment of a large number of distinct glycans to a large number of distinct non-carbohydrate moieties.

The GLYDE standard also includes a set of rules, naming conventions for the parts, and enumeration of chemical entities that are acceptable parts at various levels of granularity. These implementation rules are absolutely required for representational consistency and disambiguation, as purely syntactic enforcement of these rules (e.g. solely by the GLYDE schema) is not possible.

### History

The GLYDE format for the representation of glycan structure was developed to take advantage of the hierarchical syntax and extensibility of XML. The first version of GLYDE (Sahoo et al., 2005) used a hierarchical XML tree to intuitively mirror tree-like structures of branched glycan structures. However, this approach was found to be insufficient to represent all of the structural variation observed in glycans and glycoconjugates.

**Figure 1:**
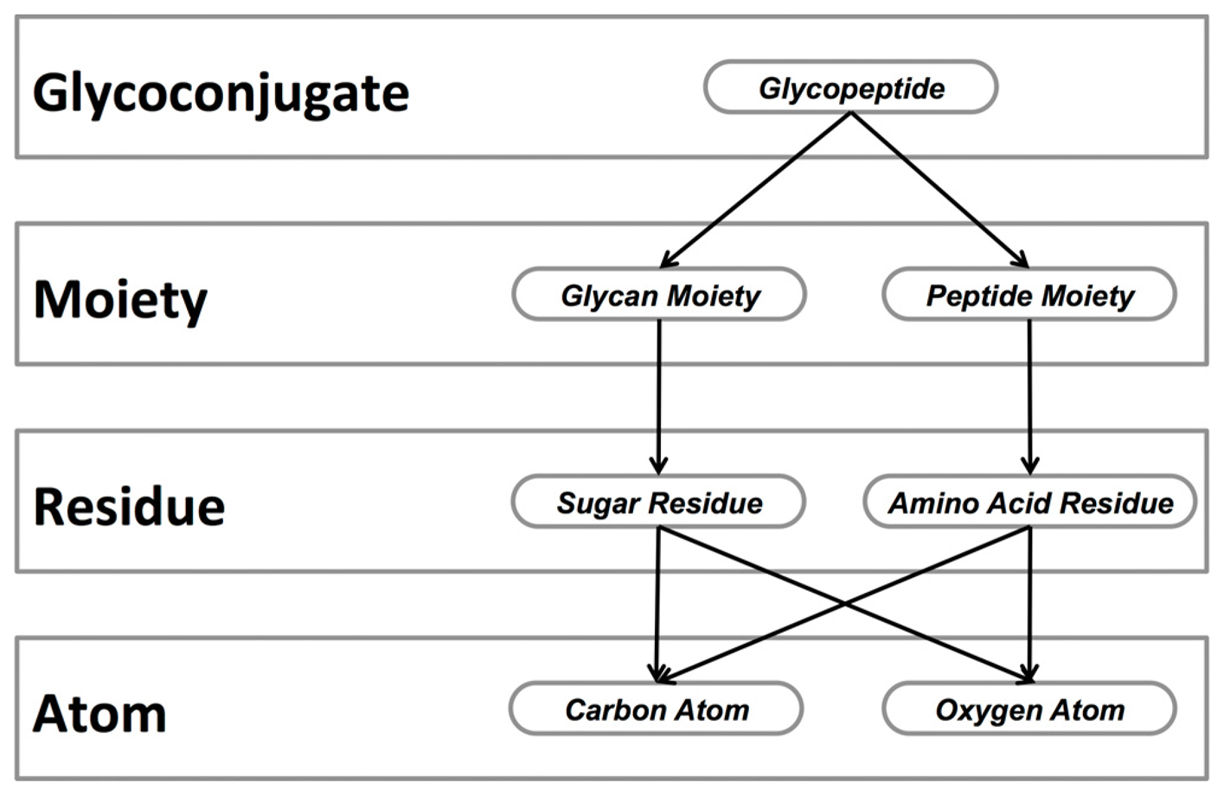
Some of the partonomy relationships implemented by GLYDE-II. Each object (rounded rectangles) is classified (as an *atom*, *residue*, etc.) according to its complexity. Arrows emanating from an object point to its parts. Each object can have many parts, only a few of which are shown. The structure of each part is defined by reference to an archetypal object. For example, the structure of each of the distinct *carbon atom* objects in a *sugar residue* is specified by reference to the archetypal *carbon atom*.

GLYDE-II was developed to account for this structural variation by using XML to implement a connection table approach. In this context, GLYDE-II provides a consistent namespace for monosaccharides and the ability to represent repeating structural features. Notably, the GLYDE-II syntax enforces a partonomic model in which structures are specified as collections of parts (Fig. 1), which can themselves be collections of smaller parts, facilitating the representation of highly complex molecules such as glycoconjugates. GLYDE-II specifies molecular topology by defining explicit connections (i.e. links) between the parts of the molecule. Version 1.2 of GLYDE introduces two important modifications: (1) classification of molecular parts using semantics that are more consistent with the way biochemists view biopolymer structure and processing and (2) optional specification of molecular geometry using a general approach that is consistent over all granularity levels and for all different classes of molecular parts. These modifications make it possible for the glycoscientist to take advantage of the GLYDE partonomy model to create and interpret representations of glycoconjugate structure that are consistent with the way biochemists think about these complex molecules and their interactions with other molecules.

### Partonomy and archetyping

One aspect that distinguishes the different glycan structure representation formats that have been developed is the way different parts of these molecules are grouped and specified. This stems from the fact that glycans are often found as components of more complex structures commonly referred to as glycoconjugates, which include glycoproteins, glycolipids and chemically modified glycans, such as oligosaccharides that have a fluorescent tag attached to the reducing end. Furthermore, the complex, branched nature of glycans has led to semantic differences in the way glycans and non-glycan moieties are described. However, GLYDE is based on semantics that are common to diverse types of biopolymers, providing an integrated way to represent the different parts of a glycoconjugate structure. This approach allows the representation and processing of structures that are specified at different levels of granularity (e.g. to identify specific components or calculate molecular masses) to be performed using a common set of algorithms. To make this practical, structural granularity must be rigorously defined using the concept of partonomy (Casati and Varzi, 1999), in which each object is described as a collection of parts (Fig. 1).

Along with partonomy, object archetyping is another core feature of GLYDE. For example, the glycan *moiety* of a glycoconjugate (e.g. glycopeptide) is defined by reference to a glycan *molecule* (e.g. the glycan released from a glycopeptide by the enzyme PNGase-F). Such use of a small archetype *molecule* to describe a part of a larger *molecule* is consistent with conventional chemical language. For example, a glucose *residue* in a glycan is customarily denoted by reference to the *molecule* glucose (a monosaccharide). These semantics are based on concepts that are both historical (e.g. the characterization of small biomolecular building blocks such as purines, sugars and amino acids by Fischer Kunz, 2002) and biochemical (e.g. the transformation of such small molecules into so-called “residues” when they are incorporated into biopolymers). This approach makes the structural representations much more concise by allowing detailed structural information (and/or references to external sources) to be specified one time for the archetype and inferred thereafter when the archetype is referenced. Archetyping atoms makes the structural representation somewhat more concise. However, archetyping larger structures (like sugar residues or glycan moieties) makes the structural representation substantially more concise. This is an important consideration when using a format such as XML that is verbose by nature.

### Implementation of the GLYDE-II partonomy model

**Figure 2:**
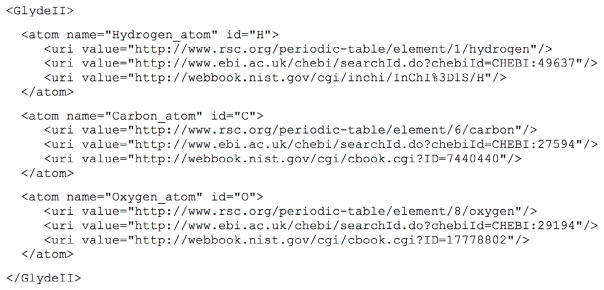
GLYDE representation of a collection of three *atom* objects (hydrogen, carbon, oxygen). External sources of information about the structure of each *atom* are indicated by *uri* tags.

A key function of GLYDE is to provide a basis for the operational classification of molecular objects, using a vocabulary indicated by *italics* in the text below. At the most fundamental level, GLYDE specifies structures as collections of atoms. Fig. 2 illustrates a minimal GLYDE-II representation of three *atom* objects. By definition, an *atom* object is not connected to another *atom* object by a molecular bond. In some cases, an unattached *atom* can be specified as a part of a GLYDE structure, but more frequently an *atom* is used as an archetype for a *bound atom*, which is, by definition, connected to at least one other *bound atom* by a covalent bond in the context of a *molecule*, which is a collection of molecular parts that are connected by covalent bonds. However, a *molecule* is defined such that it is not connected to any other *molecule* by covalent bonds.

**Figure 3:**
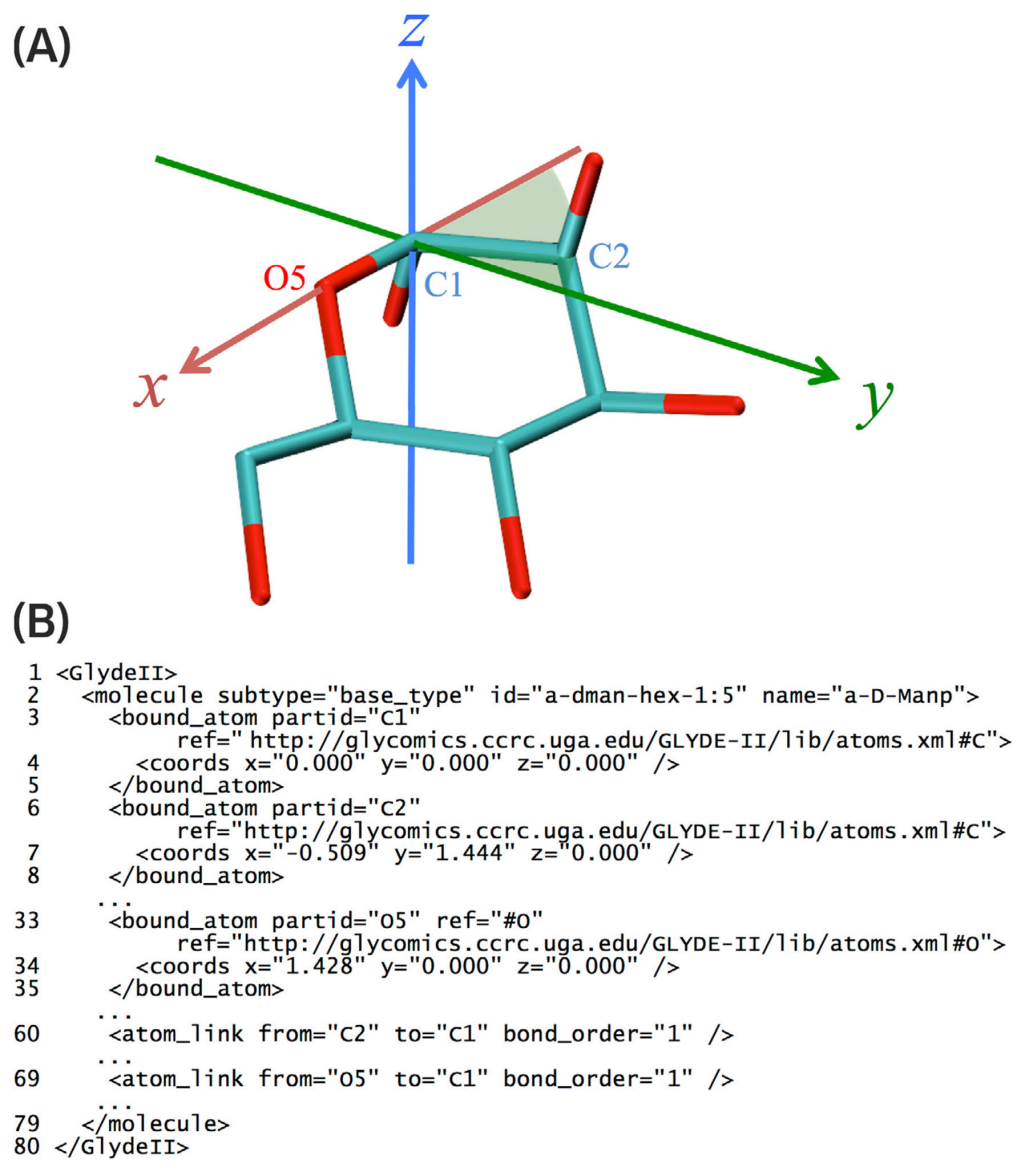
Stick model (A) and abbreviated GLYDE-II representation (B) of an α-D-Man*p molecule*. (Several lines of XML code are omitted for brevity; the remaining lines are numbered.) The structure of each *bound atom* is specified by its *ref* attribute (lines 3, 6 and 33), which points to a GLYDE-II representation of the *atom* (Fig. 2) serving as the archetype for the *bound atom*. The molecular topology is fully specified by *atom link* objects (e.g. lines 60 and 69), which connect *bound atom* objects. The molecular configuration (stereochemistry) is specified explicitly by listing the coordinates of each *bound atom*. Alternatively, stereochemistry of the *molecule* can be inferred from its *id* (line 2), which by rule corresponds to its representation using a format based on GlycoCT. The conventional orientation and position of the Cartesian axes in the atomistic GLYDE-II representation is defined by the alignment of three key *bound atom* objects: the anomeric carbon (C1 in this case) is at the origin, the ring oxygen (O5 in this case) is on the *x*-axis, and the highest-numbered carbon (C2 in this case) that is directly linked to the anomeric carbon is in the first or second quadrant of the *x*,*y*-plain (where *y* > 0).

**Figure 4:**
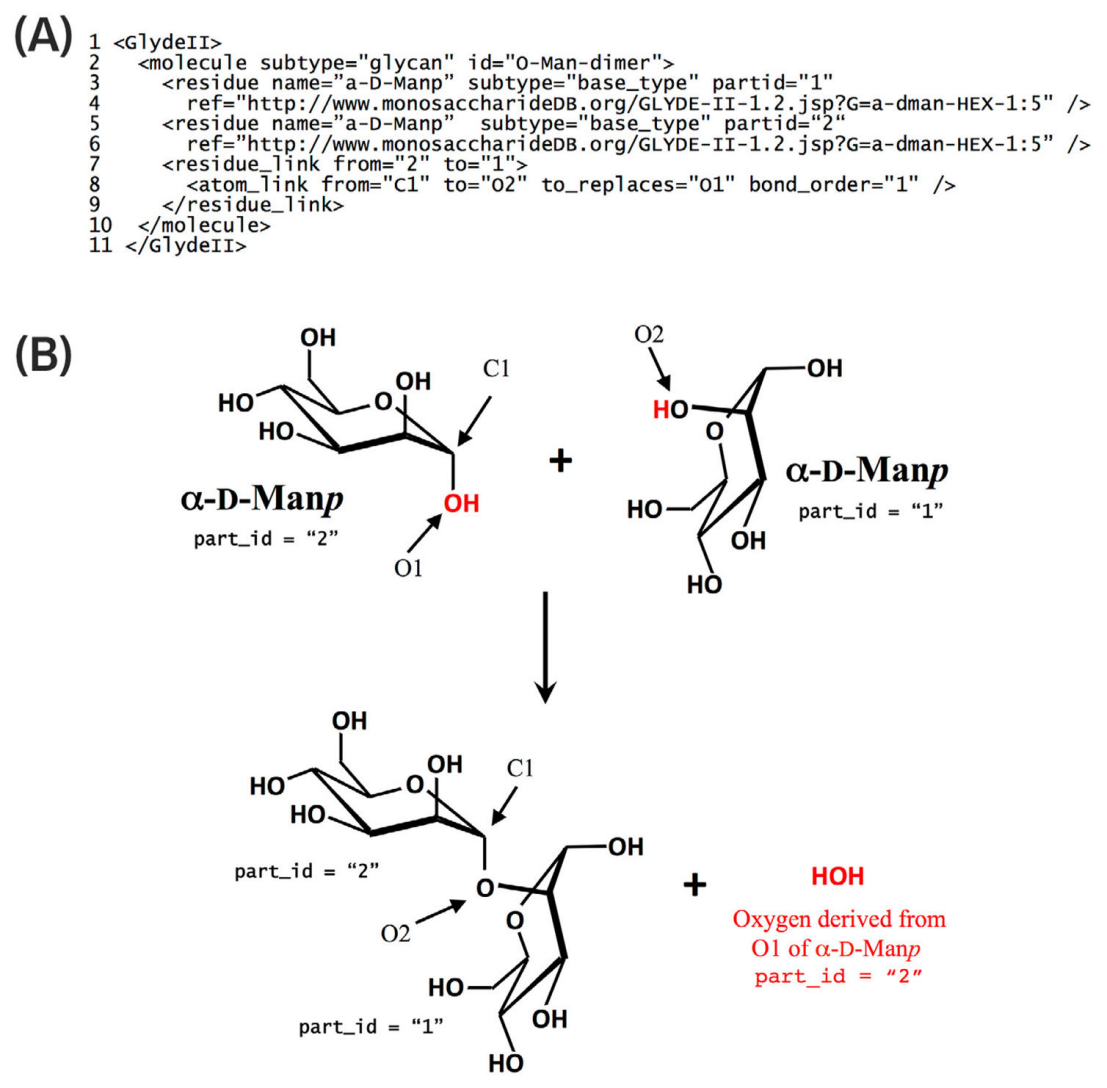
(A) GLYDE-II representation of the disaccharide *molecule* that is used as an archetype for the disaccharide *moiety* shown in Fig. 5. The properties of each of the *residue* objects in this *molecule* are specified by reference to the archetypal α-D-Man*p molecule* shown in Fig. 3. The *residue link* (line 7) formally indicates a directed bond *from* one *residue to* another. This *residue link*, in turn, encompasses an *atom link* (line 8) from C1 of one of the α-D-Man*p residue* objects (part id = “2”) to O2 of the other α-D-Man*p residue* object (part id = “1”). These directional semantics allow unambiguous interpretation of the attributes (e.g., *from* = “C1” and *to* = “O2”) in the *atom link*. That is, the bond extends from “C1” of α-D-Man*p* residue (part id = “1”) to “O2” of α-D-Man*p residue* (part id = “2”). This code also infers that, in the context of this *atom link*, “to” is a synonym for “O2 of α-D-Man*p residue* #1” and “from” is a synonym for “C1 of α-D-Man*p residue* #2”. By rule, an atom in one *residue* can replace an atom in the other *residue* when a *residue link* is formed (see Panel B). The *atom link* attribute *to replaces* = “O1” can thus be unambiguously interpreted as “O2 of α-D-Man*p residue* #1 replaces O1 of α-D-Man*p residue* #2”. (B) Formation of the glycosidic bond connecting the two α-D-Man*p residue* objects in the disaccharide encoded by the text in (A). The GLYDE convention dictates that the geometry of each archetypal monosaccharide *molecule* is retained when it is transformed into a *residue*. During this transformation, the glycosidic oxygen of one *residue* (e.g. O1 of the α-D-Man*p residue* #2) is released as a water molecule and replaced by an atom in the other *residue* (e.g. O2 of α-D-Man*p residue* #1).

In general, the GLYDE specification of a *molecule* consists of a list of its parts and the links between those parts. A *molecule* is most directly represented in GLYDE as a collection of *bound atom* objects and the *atom link* objects that connect them, as illustrated for a monosaccharide *molecule* (α-D-Man*p*) in Fig. 3. Within such a molecule representation, the structure of each *bound atom* is specified by its *ref* attribute, which indicates the *id* attribute of the *atom* object (Fig. 2) that serves as the archetype for the *bound atom*. A *molecule* can also be specified as a collection of parts that are more complex than atoms. For example, a molecule can be composed of *residue* objects (Fig. 4), whose structures are themselves specified by reference to *molecule* objects (e.g. monosaccharides), which in turn are specified as collections of *bound atom* and *atom link* objects (Fig. 3). Connections between *residue* objects are specified by *residue link* objects, which encompass *atom link* objects that specify the atoms involved in the covalent bonds connecting the *residue* objects (Fig. 4).

**Figure 5:**
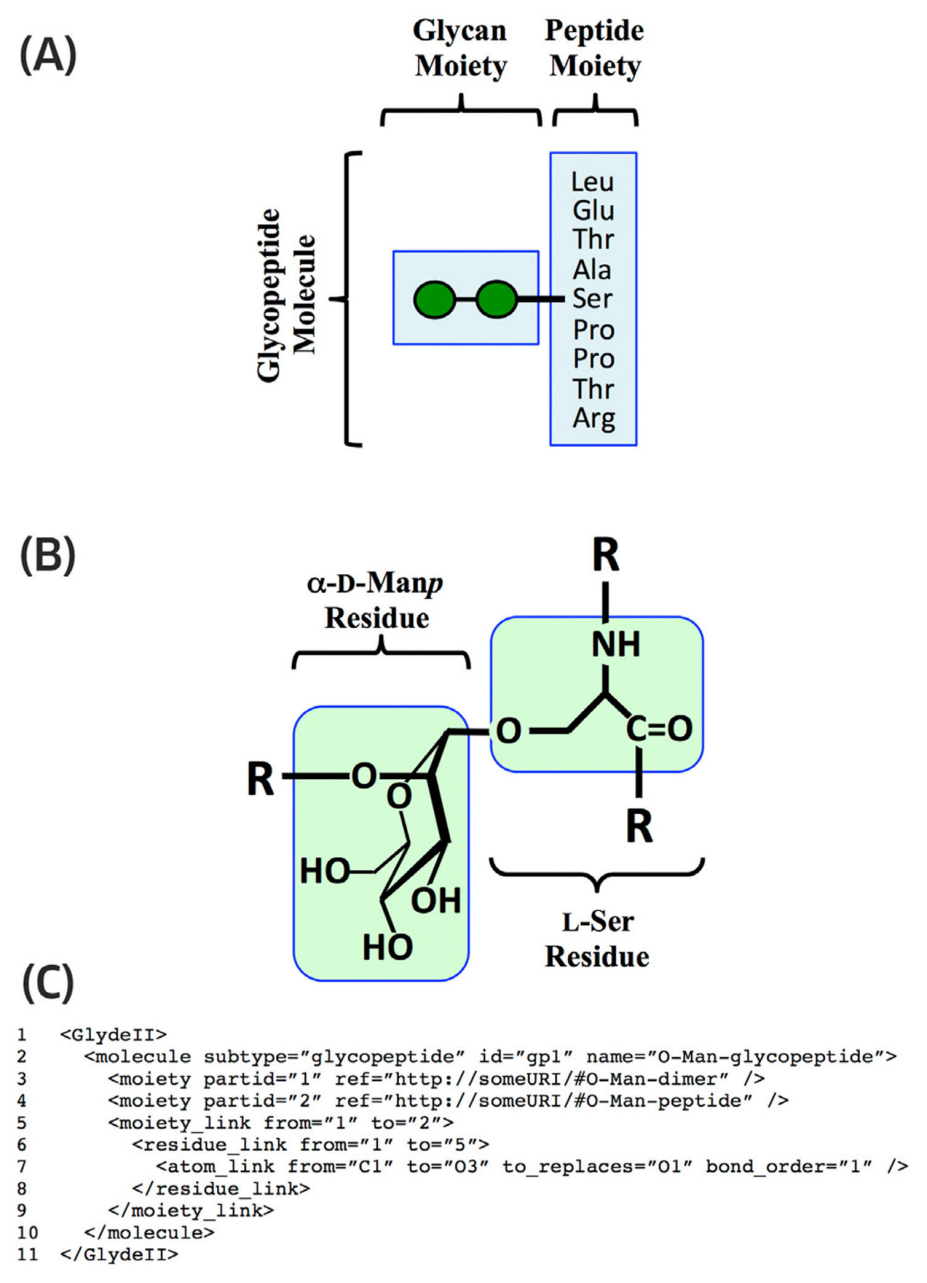
The structure of a glycopeptide *molecule* illustrated as a hierarchical collection of GLYDE parts (*bound atom*, *residue* and *moiety*) connected by links. Residues in the glycan *moiety* are represented using the CFG graphical format for glycan structure. (A) The glycan *moiety* and peptide *moiety* are connected by a *moiety link*, which embodies a *residue link* connecting one of the α-D-Man*p residue* objects (green circle) in the gly-can *moiety* to the Ser *residue* in the peptide *moiety*. (B) The two residues that connect the moieties are shown in atomic detail. The *residue link* connecting these residues embodies an *atom link* connecting C1 of the α-D-Man*p* residue to O3 of the Ser *residue*. (C) GLYDE-II representation of the glycopeptide. The *moiety link* indicates that the “O-Man-dimer” (Fig. 4) is covalently attached to the “O-Man-peptide”, whose structure is specified in “http://someURI”. The enclosed *residue link* indicates that the α-D-Man*p* residue (partid = “1” in the GLYDE-II specification of the “O-Man-dimer”) is linked to the L-Ser residue (partid = “5” in the GLYDE-II specification of the “O-Man-peptide”). The further enclosed *atom link* indicates that the *bound atom* C1 (partid = “C1” in the referenced GLYDE-II specification of α-D-Man*p* residue objects in the “O-Man-dimer” moiety) is covalently attached to the *bound atom* O3 (par-tid = “O3” in the referenced GLYDE-II specification of the L-Ser residue objects in the “O-Man-peptide”).

The structures of complex *molecule* objects such as glycoconjugates are specified using the same hierarchical approach (Fig. 5), in which larger structures are constructed as collections of smaller structures. Connections between the larger structures embody connections between the smaller structures that they contain. For example, *moiety link* objects embody *residue link* objects.

### Molecular geometry in GLYDE-II

Several different systematic nomenclatures (*R*/*S*, *α*/*β*, D/L, etc.) have been developed to specify molecular stereochemistry. However, assigning the stereochemistry of an asymmetric atom (or generating an explicit geometric interpretation of such an assignment) using a systematic nomenclature can be computationally challenging. GLYDE addresses this issue by allowing the stereochemistry of a molecule to be specified using two different methods (1) explicitly defining the three-dimensional geometry of each its parts using Cartesian coordinates; (2) parsing the *ref* attribute of each *residue* component of a *molecule*. Method (1) provides a unified way to specify the configurational geometry of all the atoms in a complex biopolymer without having to parse a systematic stereochemical nomenclature (or combination of different nomenclatures). Method (2) is much more efficient in contexts where an atomistic representation is not required. In the case of a monosaccharide *residue*, the value of the *ref* attribute is a string corresponding to its GlycoCT-based representation, which was developed as part of the EUROCarbDB initiative. This allows, for example, α-linked glucose residues to be distinguished from β-linked glucose residues in a trivial manner without any need to generate and compare atomistic representations, which are rarely required for logical inference. Thus, GLYDE fully supports both atomistic and abstract representations of molecular geometry, and notably, provides a logical and consistent framework for both representations at different levels of granularity. The conventions defined in GLYDE facilitate the implementation of algorithms to interconvert the GLYDE representation with fully atomistic representations (see Supporting information).

### Classification of parts

Judicious selection of the appropriate granularity in specifying or parsing a GLYDE representation will facilitate the development of algorithms to identify correlations between the structure of a biomolecule and its biological function or physical properties. Therefore, parts at each level of granularity are distinguished using chemical and biosynthetic criteria. For example, several different *moiety* types are defined, including *glycan moiety*, *peptide moiety* and *lipid moiety*. A glycan moiety is composed primarily of monosaccharide residues (typically connected by glycosidic bonds) while a peptide moiety is composed primarily of amino acid residues (typically connected by amide bonds). Although these semantics are not required to completely define the chemical structure of a biopolymer, they are useful for identifying chemical and computational contexts in order to facilitate the use of the structural information for data processing and knowledge discovery.

### GLYDE-II rules

The GLYDE syntax, described above, is not in itself sufficient to ensure that structural representations of glycoconjugates will be consistent. Additional rules are required to standardize parameters such as the *part id* attribute of a *bound atom* and the *id* of an archetype *molecule* (Supporting information). As mentioned above, the *id* attribute of a monosaccharide *molecule* corresponds to the GlycoCT-based representation of that molecule. This provides a convenient way to compare monosaccharide *residue* compositions or stereochemistry without generating a fully atomistic representation of the glycoconjugate. This is possible since the GlycoCT namespace for monosaccharides is machine-readable, providing unique, unambiguous names that encode the salient chemical properties of these molecules. A large collection of GlycoCT representations are maintained and curated by MonosaccharideDB (http://www.monosaccharidedb.org/). By rule, the *ref* attribute of a GLYDE monosaccharide *residue* is a uniform resource identifier (URI) consisting of the following three parts: (1) the uniform resource locator (URL) of MonosaccharideDB (“http://www.monosaccharideDB.org/”); (2) a series of characters corresponding to an http GET request (“GLYDE-II-1.2.jsp?G=“) (3) the GlycoCT string representing the molecule (e.g. “b-dglc-HEX-1:5”).

### Applications

The GLYDE-II format is currently supported in the widely used MS annotation software GlycoWorkbench as an option to export and import glycan structures. It is also being used as the standard format for common web service interfaces that are being developed to enable exchange of carbohydrate structural information among databases and software applications. These include the GlycO ontology at the CCRC, the database of the Consortium for Functional Glycomics (CFG), the Kyoto Encyclopedia of Genes and Genomes (KEGG) via the RINGS portal at Soka University (http://rings.t.soka.ac.jp/), UniCarb-DB, EUROCarbDB, the meta-database of carbohydrate structure GlycomeDB and the recently initiated GlyTouCan glycan structure registry. Support and utilization of GLYDE-II is a core feature of our Qrator software, which provides an intuitive interface that facilitates the human curation of glycan structures (Eavenson et al., 2015). We continue to develop software that fully supports the GLYDE-II format.

## Supplementary Material

GLYDE-II Supp Data

## Appendix A. Supplementary data

Supplementary data associated with this article can be found, in the online version, at http://dx.doi.org/10.1016/j.pisc.2016.05.013.
