# Supplementary material for: GLYDE-II: The GLYcan data exchange format
Source: Perspect Sci (Neth). Author manuscript; Available in PMC 2017 Sep 25. (PMC5611833; doi:10.1016/j.pisc.2016.05.013)
Supplement: GLYDE-II Supp Data [file NIHMS852483-supplement-GLYDE-II_Supp_Data.pdf]

Supporting Information

GLYDE-II: The GLYcan Data Exchange Format

Rene Ranzinger[1], Krys J. Kochut[2], John A. Miller[2], Matthew Eavenson[2], Thomas Lütteke[3] and William S. York[1,2,*]

[1]Complex Carbohydrate Research Center, University of Georgia, USA

[2]Computer Science Department, University of Georgia, USA

[3]Institute of Veterinary Physiology and Biochemistry, Justus-Liebig-University Giessen, Germany

The most up-to-date version of this document can be found at http://glycomics.ccrc.uga.edu/GLYDE-II/GLYDE-II.DTD

1. Overview.

GLYDE is a standard for the representation of the chemical structures of complex glycans that is based on a connection table formalism using XML syntax. The GLYDE standard can be divided into two conceptually distinct parts, syntax and implementation. The syntax of a GLYDE document is fully defined by its schema (either a DTD or XML schema, Section 2), which provides a framework we call PARCHMENT (PARtonomy of CHeMical ENTities). PARCHMENT allows the complete structure of biological molecules (including complex glycans) to be completely and unambiguously specified at several levels of granularity. That is, PARCHMENT is a modular approach to specify molecular structure in terms of the parts that make up the whole. It provides a very general, machine-readable format (XML) for their representation. The implementation of the GLYDE standard also includes a set of rules, naming conventions for the parts, and enumeration of chemical entities that are acceptable parts at various levels of granularity. These implementation rules are absolutely required for representational consistency and disambiguation. However, purely syntactic enforcement of these rules (e.g., solely by the GLYDE schema) would be very difficult and would probably result in an unstable standard. This document describes the XML syntax and implementation rules for GLYDE.

1.1. Partonomy and granularity. The fundamental relationship between objects in the GLYDE formalism is partonomy (also known as mereology *(Casati and Varzi 1999)*. That is, larger structures are defined by their parts. For example, a *molecule* is a complex entity that consists of parts that are connected to each other. A part can be a *moiety* (such

as a glycan moiety), a *residue* (such as a glycosyl residue or an amino acid residue) or a *bound_atom* (such as a carbon atom that is covalently linked to another atom). Two parts of a *molecule* can be connected by a *link*. It is important to emphasize that one *molecule* cannot be connected to another *molecule* by a *link*. Thus, a *molecule* is an independent entity, unlike its parts, which are linked together. Another independent entity is a *free_atom*, which is an atom that is not bound to any other atom. The third independent entity is an *aggregate*, which is itself composed of independent entities (*molecules*, *free_atoms*, and/or other *aggregates*), which are not linked to each other.

An atom (either a *free_atom* or a *bound_atom*), which is not composed of smaller parts in the GLYDE formalism, is defined in the common chemical sense and includes entities such as "Oxygen_atom" and "Carbon_atom". Larger structures are built up from smaller parts in a hierarchical manner, using independent entities (*molecule* or *free_atom*) as archetypes to specify the parts of larger entities. For example, a *free_atom* ("C", "H", "N", "O", etc.) can be referenced (used as an archetype) to specify a *bound_atom* within a *molecule*, such as a monosaccharide. This monosaccharide, in turn, can be referenced (used as an archetype) to specify a glycosyl *residue* that is a part of a glycan *molecule*. This glycan *molecule*, in turn, can be referenced (used as an archetype) to specify a glycosyl *moiety* that is part of a glycoconjugate *molecule*.

At a coarser granularity, a *molecule*, which by definition is not covalently attached to any other entity, can be referenced to specify the structure of a *molecule_instance* that is one of the parts of an *aggregate*. For example, the monomeric form of avidin is a glycoprotein *molecule*, while the native form of avidin is an *aggregate* composed of four avidin *molecule_instance* objects, each specified by reference to a single avidin *molecule* object. The atomic components of an *aggregate* object are specified as *free_atom_instance* objects and the aggregate components of larger, inclusive *aggregate* objects are specified as *aggregate_instance* objects. Thus, the "instance" objects that comprise a properly constructed aggregate are defined indirectly (by reference). Such "instance" objects have additional optional attributes - Cartesian *coords* and *euler* angles, which specify the location and orientation of the instance within the aggregate. (See Section 4.) This approach facilitates the description (including geometry) of complex *aggregate* structures that contain more than one copy of each component.

In common biochemical language, a residue is often defined as a structural subunit of a biological molecule that is released by a hydrolytic chemical reaction. Thus, a GLYDE *residue* may correspond to a glycosyl residue (such as β-D-Glc*p* or α-L-Fuc*p*), an amino acid residue (such as L-Gly or L-Asn) or a lipid residue (such as oleic acid). A GLYDE *moiety*, such as a glycosyl moiety or peptide moiety within a glycopeptide, is specified by reference to a *molecule* that is composed of at least one *residue*. It is important to note that the *moiety* objects that comprise a glycopeptide are not themselves *molecule* objects, otherwise linking them to each other would violate GLYDE-II syntax. Rather, each *moiety* is a distinct instance that is defined by reference to a glycan or peptide *molecule*.

The GLYDE hierarchy thus allows structures to be represented at several different levels of granularity. For example, a software application may only require information specifying that the molecule of interest contains carbohydrate *moiety* ***X*** and peptide

moiety ***Y***, and may not depend on the molecular details of these structures. In this case, a very coarse granularity will suffice, and it will not be necessary to parse the GLYDE representation to the atomic level. A fully atomistic representation that does not provide for abstraction of larger substructures would not be appropriate for such a case, as this would require the larger substructures (e.g., moieties) to be identified and abstracted by the software application itself.

2. The GLYDE syntax - DTD.

The syntactic aspects of structure representation in GLYDE are defined by the Document Type Definition (DTD - http://www.w3.org/TR/xml/#sec-prolog-dtd). The DTD defines XML ***elements*** (http://www.w3.org/TR/xml/#elemdecls) that make up the GLYDE file. For example, the DTD specifies that a GLYDE document contains XML ***elements*** called *molecule* and *residue*. Each XML ***element*** can have several ***attributes*** (http://www.w3.org/TR/xml/#attdecls). The most recent version of the DTD file for GLYDE-II can be found at http://glycomics.ccrc.uga.edu/GLYDE-II/GLYDE-II.DTD. GLYDE is also supported using an XML schema (http://www.w3.org/XML/Schema).

While, the major features of the DTD are documented internally, additional notes are included below.

- The ***root element***, called *GlydeII*, can contain ***elements*** called *free_atom, molecule, and aggregate*. Each of these is an independent object (not covalently linked to other objects).

- When a particular *free_atom*, *molecule* or *aggregate* is present more than once (or the location and orientation of the object is important), the objects are contained in an *aggregate* object and structurally specified by reference to an archetypal object. An aggregate is simply a way to collect independent parts into a set and specify their relative positions and orientations. For example, a multimeric glycoprotein along with small ions such as Na^+ would be specified as a GLYDE-II *aggregate*.

- The *molecule* ***element*** is composed of parts and links that connect them. The DTD enforces the restriction that a *molecule* must be made entirely of *moieties*, *residues*, or *bound_atoms*, and these different types of parts cannot be mixed together. Furthermore, the links in the *molecule* must correspond to the parts. For example, a *molecule* composed of *residues* must only contain *residue_link* objects, which themselves can enclose *atom_link* objects. It is important to note that *molecules* always serve as archetypes for the polyatomic parts called *moiety, residue* and *molecule_instance*.

- The *free_atom* ***element*** has no parts, and must contain at least one *uri* ***element*** pointing to an external description of its structure.

- The *moiety*, *residue*, and *bound_atom* ***elements*** are building blocks from which *molecules* are constructed. Two *moiety* ***elements*** can only be connected by a *moiety_link*, which can wrap a residue_link that connects the two *residues* (one in

each *moiety*) involved in the *moiety_link*. Similarly, the *residue_link* can wrap an *atom_link* that connects the two *bound_atoms* (one in each *residue*) that are involved in the *residue_link*. The DTD enforces syntax that help to maintain this hierarchy and assure that each link will be appropriate and symmetrical (i.e., *moiety* to *moiety*, *residue* to *residue*, or *bound_atom* to *bound_atom*).

- Instantiation of a part of a *molecule* (i.e., a *moiety, residue, or bound_atom*) involves the specification of a *partid* ***attribute***, which is of type ***CDATA***. A partid can thus be any text, and need not be unique within a GLYDE-II file. This makes it possible to reuse the same *partid* for different *parts* in the same GLYDE file. For example, two different monosaccharide *molecules* (say "b-D-Glcp" and "b-D-Galp") in the same GLYDE file may both contain a *bound_atom* with a *partid* whose value is "C1", so this value would not be unique within the GLYDE file. Thus, while distinct elements can have the same partid in a give GLYDE-II file, each direct child of a *molecule* must have a distinct partid, such that the partid attribute can be subsequently used as an unambiguous reference to each distinct part. For example, a b-D-Glcp *molecule* can have only one bound_atom with partid="C1". Thus, one can explicitly refer to "C1" of the b-D-Glcp *molecule* (e.g., using x-path) because it is identified as the *part* with *partid*="C1" that is found within the *molecule* having *id*="b-dglc-hex-1:5". (See section 3.2 for conventions for *id* ***attributes*** of carbohydrate residues.)

- A *combination* ***element*** is a collection of links that can be combined in different ways to generate several mutually exclusive chemical entities. The combination element is used when there is ambiguity regarding the location of a part or group of parts within a *molecule*, as described in Section 3.4.4.

3. Implementation rules for GLYDE structures.

The smallest possible structures are atoms (*free_atoms* or *bound_atoms*), as illustrated in the following example.

```
<?xml version="1.0" encoding="ISO-8859-1"?>
<!DOCTYPE GlydeII SYSTEM "http://glycomics.ccrc.uga.edu/GLYDE-II/GLYDE-II-1.2.DTD">
<GlydeII>
 <atom name="Hydrogen_atom" id="H">
   <uri value="http://www.rsc.org/periodic-table/element/1/hydrogen"/>
   <uri value="http://www.ebi.ac.uk/chebi/searchId.do?chebiId=CHEBI:49637"/>
   <uri value="http://webbook.nist.gov/cgi/inchi/InChI%3D1S/H"/>
 </atom>
 <atom name="Carbon_atom" id="C">
   <uri value="http://www.rsc.org/periodic-table/element/6/carbon"/>
   <uri value="http://www.ebi.ac.uk/chebi/searchId.do?chebiId=CHEBI:27594"/>
   <uri value="http://webbook.nist.gov/cgi/cbook.cgi?ID=7440440"/>
 </atom>
 <atom name="Oxygen_atom" id="O">
   <uri value="http://www.rsc.org/periodic-table/element/8/oxygen"/>
   <uri value="http://www.ebi.ac.uk/chebi/searchId.do?chebiId=CHEBI:25805"/>
   <uri value="http://webbook.nist.gov/cgi/cbook.cgi?ID=17778802"/>
 </atom>
 <atom name="Nitrogen_atom" id="N">
   <uri value="http://www.rsc.org/periodic-table/element/7/nitrogen"/>
   <uri value="http://www.ebi.ac.uk/chebi/searchId.do?chebiId=CHEBI:25555"/>
   <uri value="http://webbook.nist.gov/cgi/inchi/InChI%3D1S/N"/>
 </atom>
</GlydeII>
```

Note that he URL of the DTD for GLYDE is indicated in the `<!DOCTYPE>` tag. The physical properties of each atom described in this file can be found by reference to the *uri* objects.

3.1. Implementation rules for atomic *structures* and *parts*. Rules (outside the DTD specification) are defined to enforce vocabulary control for atomic *structures* and *parts*.

Rule 1: The values of the *id* ***attribute*** of *free_atom* ***elements*** in GLYDE are limited to the standard elemental or isotopic string representations, such as "H", "C", "13C", etc.

Rule 2: Atomic *ids* cannot be assigned to non-atomic *structures* in a GLYDE document, as this could result in degeneracy of the *id*, which is not allowed.

Rule 3: The values of the *partid* ***attribute*** for *bound_atom* ***elements*** follow specific guidelines (described below). The reason for this rule is that it allows an application to generate GLYDE representations of a glycan without explicitly looking up the *partids* of the constituent atoms of each residue in order to assign an inter-residue link between two atoms. For example, a monosaccharide archetype for a *residue* is composed of *bound_atoms*, with *partids* "C1", "O1", etc. According to this rule, a "1-4" (*O*-glycosidic) linkage from an aldosyl residue and another monosaccharide residue is always specified by declaring: `from = "C1"` and `to = "O4"`.

The *partids* for *bound_atoms* in a carbohydrate *residue* (where `type="base_type"`) are assigned using the numbering system sanctioned by the IUPAC-IUB Joint Commission on Biochemical Nomenclature (JCBN), as described in "Symbols for Specifying the Conformation of Polysaccharide Chains" (http://www.chem.qmul.ac.uk/iupac/misc/psac.html#130). For example, "Atoms are thus designated C3, O2, H4, etc." This IUPAC-IUB document also indicates that oxygens within a furanose or pyranose ring are named using a number rather than the letter "R". Thus, the oxygen within the ring of Glc*p* is identified as "O5" rather than "OR". Exchangeable hydrogens of hydroxyl groups are not explicitly named in GLYDE. However, the GLYDE standard differs from the IUPAC standard in that prochiral atoms are indicated using the R/S nomenclature (e.g., "H6R" and "H6S"), as this is practical when specifying the stereochemistry of a well-defined small molecule such as a monosaccharide.

The atomic *partids* for amino- acids are assigned using the numbering system sanctioned by the IUPAC-IUB Joint Commission on Biochemical Nomenclature (JCBN), as described in "Nomenclature and Symbolism for Amino Acids and Peptides (3AA-2)" (http://www.chem.qmul.ac.uk/iupac/AminoAcid/AA1n2.html#AA22).

3.2. Implementation rules for monosaccharide *molecules* and *residues*. The scope and identities of monosaccharide *molecule*s and corresponding monosaccharide *residues* are

defined for GLYDE-II according to the Glyco-CT specification (Herget, Ranzinger et al. 2008). The following objects are defined:

base-type: a description of a stereo-chemically defined structure from the chemical class of polyhydroxyaldehydes or ketones, without any substituent. On this level, acidic functions, double bonds, deoxigenations, sp^2-hybridisation, reductions of the anomeric carbon, and additional carbonyls (e.g., keto groups) are encoded. Thus, base-types are polyhydroxy structures composed entirely of C, H and O atoms. This includes many common simple sugars such as glucose, mannose, and fucose. Valid base-types that contain up to four stereo-centres are named using IUPAC nomenclature (*IUPAC Nomenclature of carbohydrates, http://www.chem.qmul.ac.uk/iupac/2carb/02.html#0222*) with a single configuration specifier. For example, "dglc" signifies the D-*gluco* configuration. Base-types with more than four stereo-centres also follow established IUPAC naming convention, which results in composite names, such as "dgro-dgal" for D-*glycero*-D-*galacto* **base-types**. Trivial names for such structures are deprecated. The anomeric configuration of the **base-type** is also specified.

Substituent: A non-base-type entity with linkage(s) to a base-type. A substituent is typically a small chemical entity, which is encoded in a list of subsitutents. The MonosaccharideDB (http://www.monosaccharideDB.org) manages this list.

Monosaccharide: Every distinct *residue* entity that is connected via a glycosidic linkage to another entity – typically a **base-type** with **substitutions**.

GLYDE *residue* objects are specified by reference to *molecule* objects. For example, glycosyl residues are specified by reference to the corresponding free monosaccharide *molecule* objects. Thus, the "b-D-Glcp" **monosaccharide** *residue* corresponds to a single **base-type** and is specified by reference to the "b-D-Glcp" *molecule* as its archetype. However, many traditionally defined **monosaccharides** can be composed of a **base-type** and **substituents**. As specified by GlycoCT, m*onosaccharides* containing an *N*-acetyl group are represented by two GLYDE-II residues, a base-type and a substituent. For example, a "b-D-GlcpNAc" **monosaccharide** consists of a "b-D-Glcp" *residue* (`subtype="base_type"`) and an "n-acetyl" *residue* (`subtype="substituent"`).

By convention, the *residue_link* is from the *substituent* to the *base_type*. In the GLYDE-II representation of b-D-GlcpNAc (described above) the nitrogen of the n-acetyl *substituent* (the "from" residue) replaces O2 of the *molecule* (the monosaccharide b-D-Glcp) acting as the archetype for the b-D-Glcp *base_type* (the "to" residue), so the attribute `from_replaces="O2"` is used. See section 3.3 for more details.

Rule 4. In GLYDE, composite **monosaccharide** *molecules* that contain both a **base-type** *residue* and **substituent** residues can be defined by combining these *residues*. However, such composite **monosaccharide** *molecules* should never be referenced to specify a **monosaccharide** *residue* within the context of a larger *molecule*. That is, a composite **monosaccharide** *residue* must be explicitly defined by explicit reference to the molecules corresponding to its component parts.

<u>Rule 5.</u> The valid names for all **monosaccharides** (including composite **monosaccharides** consisting of both a **base-type** and **substituents**) are managed by the MonosaccharideDB (http://www.monosaccharideDB.org), which also manages the naming of **base-types** and **substituents** as well as the naming of atoms in these structures. MonosaccharideDB provides several services. For example, it allows a **monosaccharide** to be identified from its **base-type** and **substituents**, and provides alternate names (IUPAC, trivial) of the **monosaccharide**.

<u>Implementation Note.</u> To maintain vocabulary consistency, GLYDE representations of monosaccharide *molecules* that are used as archetypes for carbohydrate *residues* will be dynamically generated by services provided by MonosaccharideDB. Thus, full specification of the *ref* attribute of a *part* will include the MonosaccharideDB URL and the Glyco-CT name of the *part*. For example,

```
ref="http://www.monosaccharideDB.org/GLYDE-II.jsp?G=a-dman-hex-1:5"
```

The GLYDE representation can be made more concise by using a DTD ***entity*** (http://www.w3.org/TR/xml/#sec-references) to define the URL of the service provided by MonosaccharideDB. For example, the GLYDE file could contain the following code:

```
<!DOCTYPE GlydeII SYSTEM "http://glycomics.ccrc.uga.edu/GLYDE-II/GLYDE-II-1.2.DTD "[
 <!ENTITY mDBget "http://www.monosaccharideDB.org/GLYDE-II.jsp?G">
]>
```

Then, reference to a **base-type** or **substituent** in MonosaccharideDB can be succinctly made using the code like the following example:

```
ref="&mDBget;=a-dman-hex-1:5"
```

Subsequent examples in this document will use this succinct representation. In the future, the ***entity*** specifying the URL of the MonosaccharideDB service used in this context will be included in the DTD or XML schema itself, to insure that MonosaccharideDB is used as the authority for defining residue *partid* attributes.

Currently, a subset of GLYDE-II conformant atomistic representations of **base-types** and **substituents** are available via MonosaccharideDB. This set will be expanded in the future.

3.3. Implementation rules for the direction of links. The GLYDE standard includes a partonomy of links. (See Section 1.1.) That is, a *residue_link* between two *residues* (e.g., specifying linkage of residues “A” and “B”) embodies an *atom_link* with finer granularity (e.g., specifying linkage of “C1” of residue “A” and “O4” of residue “B”). This is implemented using the *from* and *to* ***attributes*** of each link to specify an <u>ordered pair</u>. Thus, the object specified by the *from* ***attribute*** of a child link corresponds to a part of the

object specified by the "*from*" **attribute** of the parent *link*. A similar relationship applies to the "*to:* ***attribute***. In order to maintain consistency in the specification of structures, rules are required to constrain the ordering of this pair (i.e., which of two parts is specified by the *from* ***attribute*** and which is specified by the *to* ***attribute***). In general, one should be able to trace at least one pathway between any part of the structure and its root without reversal of link direction. (However, it is recognized that one might find structures for which no set of specific rules can be specified that maintain this general rule.)

Note that the rules specifying the direction of links do not apply to the order that parts are listed in the file, as GLYDE-II makes no effort to generate unique representations, where the text order is controlled.

> ***Rule 6.*** Direction of *atom_links*. The *atom_links* between two atoms in a monosaccharide all point toward the attached carbon with the lowest number, (e.g., from C2 to C1, from O2 to C2, from O1 to C1, etc). The *atom_link* between the anomeric carbon and the ring oxygen is an exception. That is, the *atom_links* around the ring all point in the same direction, so the *atom_link* connecting C1 and O5 in glucopyranose is from C1 to O5. The *atom_links* between two atoms in an amino acid all point toward C1 (the carbonyl carbon that can take part in a peptide linkage). The *link* between an atom in one residue and an atom in another residue follow from ***Rule 7***.

> ***Rule 7.*** Direction of *residue_links*. The *residue_links* between glycosyl residues point toward the reducing end of a glycan. The *residue*_links between amino acid residues point toward the carboxy terminus. The *residue*_links between a monosaccharide residue and an amino acid (as in a glycopeptide) point toward the amino acid if the link is via a glycosidic bond and toward the glycosyl residue if the link is via an ester involving the carboxyl of the amino acid and a hydroxyl of the monosacharide residue. That is, ester and amide *residue_links* generally point from the acid-containing *residue* to the alcohol- or amine-containing *residue*, respectively. An exception is the link from the sugar to the peptide in an N-glycopeptide, where the link is from the sugar residue to the asparagine residue.

> ***Rule 8.*** All links must connect two parts that are of the same granularity. The "*from*" and "*to*" ***attributes*** of the child link point to structures that have a granularity that is exactly one level finer than those in the parent link. That is, an *atom_link* is always the child of a *residue_link*, which is always the child of a *moiety_link*. (In *molecule* objects such as monosaccharides that are directly composed of *bound_atom* objects, the *atom_link* has no parent. In *molecule* objects such as free glycans that are directly composed of *residue* objects, the *residue_link* has no parent. Only when the *molecule* is used as an archetype for a *residue* or *moiety* are these links wrapped by links of higher granularity.)

These rules for GLYDE-II are illustrated in the following example, drawn using the CFG graphical nomenclature. The *part_id* of each *residue* is labeled with a number. GlcpNAc residues are composed of a *base_type* residue and a *substituent* residue.

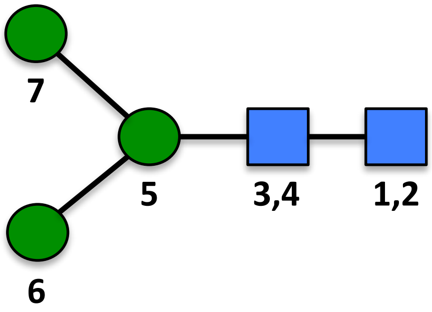


```
<?xml version="1.0" encoding="ISO-8859-1"?>
<!DOCTYPE GlydeII SYSTEM "http://glycomics.ccrc.uga.edu/GLYDE-II/GLYDE-II-1.2.DTD"[
 <!ENTITY mDBget "http://www.monosaccharideDB.org/GLYDE-II.jsp?G">
]>
<GlydeII>
 <molecule subtype="glycan" id="M3N2">
  <residue subtype="base_type" partid="1" ref="mDBget;=b-dglc-HEX-1:5" />
  <residue subtype="substituent" partid="2" ref="mDBget;=n-acetyl" />
  <residue subtype="base_type" partid="3" ref="mDBget;=b-dglc-HEX-1:5" />
  <residue subtype="substituent" partid="4" ref="mDBget;=n-acetyl" />
  <residue subtype="base_type" partid="5" ref="mDBget;=b-dman-HEX-1:5" />
  <residue subtype="base_type" partid="6" ref="mDBget;=a-dman-HEX-1:5" />
  <residue subtype="base_type" partid="7" ref="mDBget;=a-dman-HEX-1:5" />
  <residue_link from="2" to="1">
   <atom_link from="N1" to="C2" from_replaces="O2" bond_order="1" />
  </residue_link>
  <residue_link from="3" to="1">
   <atom_link from="C1" to="O4" to_replaces="O1" bond_order="1" />
  </residue_link>
  <residue_link from="4" to="3">
   <atom_link from="N1" to="C2" from_replaces="O2" bond_order="1" />
  </residue_link>  <residue_link from="5" to="3">
   <atom_link from="C1" to="O4" to_replaces="O1" bond_order="1" />
  </residue_link>
  <residue_link from="6" to="5">
   <atom_link from="C1" to="O3" to_replaces="O1" bond_order="1" />
  </residue_link>
  <residue_link from="7" to="5">
   <atom_link from="C1" to="O6" to_replaces="O1" bond_order="1" />
  </residue_link>
 </molecule>
</GlydeII>
```

Application note. In this example, evaluation of the ***entity*** “`&mDBget;`” represents the URL that points to a MonosaccharideDB, instructing it to dynamically generate the GLYDE-II representations of an archetypal *molecule*. However, it is possible that some *molecules* (e.g., oligosaccharides that are used as archetypes for the carbohydrate *moieties* of glycoconjugates) or *free_atoms* (that are used as archetypes for *bound_atoms*) will be found in static XML files. In other cases, the archetypal *molecules* or *free_atoms* may reside in the same XML file as the parts that reference them. In any case, a GLYDE parser must know where to look for the referenced *molecule* or *free_atom* within the GLYDE code. This is accomplished by dividing the string specified by the *ref* ***attribute*** into two substrings separated by a delimiter. For static representations “#” is used as the delimiter. For dynamically generated representations, “=” is used as the delimiter. The substring following the

delimiter is the *id* ***attribute*** of the *molecule* or *free_atom* that is being references as an archetype for the part.

In summary, if the structure is found in the same XML file as the part that references it, “#” is the first character in the string specified by the *ref* ***attribute*** of the *part*, as follows.

```
<moiety subtype="glycan" partid="moiety_1" ref="#M3N2"/>
```

If the structure is found in a different XML file, the “#” delimiter is used as follows.

```
<moiety subtype="glycan" partid="moiety_1"
   ref="http://glycomics.ccrc.uga.edu/GLYDE-II/lib/M3N2.xml#M3N2"/>
```

If the structure is found in a dynamically generated GLYDE-II representation, the “=” delimiter is used as follows (see Application Note paragraph, above).

```
<residue subtype="base_type" partid="3" ref="&mDBget;=b-dglc-hex-1:5"/>
```

It is important to note that when the archetype *molecule* with `id="b-dglc-hex-1:5"`, for example, is used as a part in a larger *molecule*, it is recast as a *residue*. That is, the original *moleule* is an independent entity, the monosaccharide “β-D-Glcp”. However, within the context of a larger *molecule*, this part is not independent (it is glycosidically linked), and therefore it is no longer an independent monosaccharide *molecule*, but a *residue* within a glycan *molecule*.

Each of the links in this pentaglycoside is specified at two levels of granularity. The coarsest level (a *residue_link*) just specifies that two residues are connected to each other. Each of the *residue_links* has a child *atom_link* that embodies a finer granularity. For example, the following code snippet

```
<residue_link from="6" to="5">
 <atom_link from="C1" to="O3" to_replaces="O1" bond_order="1" />
</residue_link>
```

specifies that there is a *link* from the residue with `partid="6"` *to* the residue with `partid="5"`, and that this link is actually a covalent bond from “C1” of the residue with `partid="6"` to “O3” of the residue with `partid="5"`. The strings “C1” and “O3” are *partid* **attributes** of *bound_atom* objects in the GLYDE-II representations of the archetype molecules specifying the structures of the “*from*” residue (`partid="6")` and the “*to*” residue (`partid="5"`), respectively. Furthermore, the ***attribute*** `to_replaces="O1"` specifies that the atom **to** which the bond extends (i.e., “O3” of the residue with `partid="5"`) **replaces** “O1” of the residue **from** which the bond extends (i.e, the residue with `partid="56"`). This reflects the chemistry of glycosidic-bond formation, which transforms the monosaccharide *molecule* into a *residue*. As illustrated in the Figure below, bond formation is a dehydrating condensation that results in the liberation of a molecule of water. In the example above, the oxygen of this liberated water molecule is derived from “O1” of the *residue* with `partid="6"`, and this oxygen is replaced by “O3” of the *residue* with `partid="5"` when the bond is made. (The two

hydrogen atoms of the liberated water are not specified in the definition of the residues, as they are exchangeable hydrogens that are only transiently attached to oxygen atoms.)

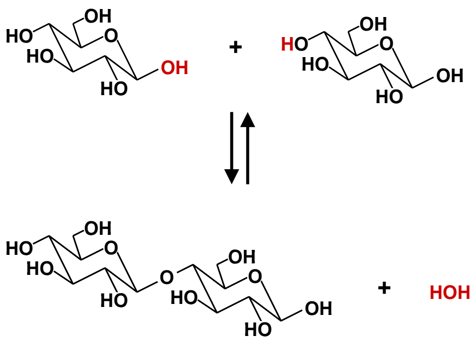

Glycosidic bond formation is a dehydrating condensation

One might imagine designing a carbohydrate *residue* archetype that does not contain "O1", making it unnecessary to specify the replacement of this oxygen upon glycosidic bond formation. However, this approach would have several key disadvantages. (1) It presumes that all bond formations in which the residue participates involve the loss of O1, which may not be true. (2) Failure to include O1 in the definition of the carbohydrate residue makes it difficult, if not impossible, to specify the stereochemistry (anomeric configuration - α or β) of C1 using a formalism that specifies the parity of the anomeric carbon in the archetype. (3) Leaving out O1 in the definition of the carbohydrate *residue* forces one to add an oxygen atom (O1) in order to use this structure as an archetype to represent a reducing *residue*. In summary, it is critical to have the ability to specify that bond formation by chemical condensation results in the replacement of a specific atom(s) in the newly linked residue(s). This is also true when specifying links between amino acids in peptides, where bond formation results in the loss of one of the carboxylate oxygens of the amino-acid *residue* from which the bond extends. Finally, this approach is consistent with atom replacement formalism of Glyco-CT (Herget, Ranzinger et al. 2008)

Rule 8 requires that, when the glycosidic part of a glycocongugate consists of a single *residue* (such as an α-D-Man*p* residue linked to a serine residue in a protein), that singel residue must be "wrapped" in the (monoglycosyl) *moiety* that contains it. The *molecule* used as the archetype for this *moiety* must contain a single α-D-Man*p residue*. Directly linking the α-D-Man*p residue* to the peptide *moiety* would break ***Rule 8***, making it difficult to implement code to parse the connection between objects. In other words, a three-level link is required in this case: (i) from the monosaccharide *moiety* to the peptide *moiety*; (ii) from the α-D-Man*p residue* to the serine *residue*; and (iii) from C1 (a *bound_atom*) of the α-D-Man*p* residue to O3 (a *bound_atom*) of the serine residue. The code on the next page illustrates such a structure and the combination of several structural entities into a single GLYDE-II file.

```
<?xml version="1.0" encoding="ISO-8859-1"?>
<!DOCTYPE GlydeII SYSTEM "http://glycomics.ccrc.uga.edu/GLYDE-II/GLYDE-II-1.2.DTD"[
 <!ENTITY mDBget "http://www.monosaccharideDB.org/GLYDE-II.jsp?G">
]>
<GlydeII>
  <molecule subtype="glycan" id="glycan_1" name="monoglycosyl glycan">
   <residue subtype="base_type" partid="man_1" ref="&mDBget;=a-dman-HEX-1:5"/>
  </molecule>

  <molecule subtype="peptide" id="peptide_1" name="dipeptide">
   <residue subtype="amino_acid" partid="ser_2" ref="&mDBget;=lser"/>
   <residue subtype="amino_acid" partid="gly_1" ref="&mDBget;=lgly"/>
   <residue_link from="gly_2" to="ser_1">
     <atom_link from="C1" to="N2" to_replaces="O1"/>
   </residue_link>
  </molecule>

  <molecule id="gp1" name="mannosylated peptide">
   <moiety subtype="glycan" partid="moiety_1″ ref="#glycan_1"/>
   <moiety subtype="peptide" partid="moiety_2" ref="#peptide_1"/>
   <moiety_link from="moiety_1" to="moiety_2">
     <residue_link from="man_1" to="ser_2">
      <atom_link from="C1" to="O3" to_replaces="O1"/>
     </residue_link>
   </moiety_link>
  </molecule>
</GlydeII>
```

Detail. The hierarchical structure of the *moiety_link* in the GLYDE-II representation of the mannopeptide, showing a covalent bond *from* [“C1” of “man_1” of “moiety_1”] to [“O3” of “ser_2” of “moiety_2”]. Within the *atom_link*, “to” is assigned as a synonym for “O3”, such that `to_replaces="O1″` indicates that “O3” of “ser_2” replaces “O1” of “man_1”. That is, the *to_replaces* ***attribute*** directly specifies the atom in the “from” residue that is replaced by the “to” atom, which is indirectly specified by the *to* ***attribute***.

3.4. Atypical structures. GLYDE-II is capable of representing atypical structures, such as cyclic glycans and large glycans with repeating structures.

3.4.1. Macrocyclic structures. The connection-table format of GLYDE-II makes representation of fully defined cyclic structures trivial, as illustrated in the following example, where simply adding a *residue_link* from residue “1” to residue “6” cyclizes the molecule.

```
<?xml version="1.0" encoding="ISO-8859-1"?>
<!DOCTYPE GlydeII SYSTEM "http://glycomics.ccrc.uga.edu/GLYDE-II/GLYDE-II-1.2.DTD"[
 <!ENTITY mDBget "http://www.monosaccharideDB.org/GLYDE-II.jsp?G">
]>
<GlydeII>
  <molecule subtype="glycan" id="a-cd" name="alpha-cyclodextrin">
   <residue subtype="base_type" partid="1" ref="&mDBget;=a-dglc-hex-1:5"/>
   <residue subtype="base_type" partid="2" ref="&mDBget;=a-dglc-hex-1:5"/>
   <residue subtype="base_type" partid="3" ref="&mDBget;=a-dglc-hex-1:5"/>
   <residue subtype="base_type" partid="4" ref="&mDBget;=a-dglc-hex-1:5"/>
   <residue subtype="base_type" partid="5" ref="&mDBget;=a-dglc-hex-1:5"/>
   <residue subtype="base_type" partid="6" ref="&mDBget;=a-dglc-hex-1:5"/>

   <residue_link from="2" to=" 1">
     <atom_link from="C1" to="O4" to_replaces="O1" bond_order="1"/>
   </residue_link>
   <residue_link from="3" to="2">
     <atom_link from="C1" to="O4" to_replaces="O1" bond_order="1"/>
   </residue_link>
   <residue_link from="4" to="3">
     <atom_link from="C1" to="O4" to_replaces="O1" bond_order="1"/>
   </residue_link>
   <residue_link from="5" to="4">
     <atom_link from="C1" to="O4" to_replaces="O1" bond_order="1"/>
   </residue_link>
   <residue_link from="6" to="5">
     <atom_link from="C1" to="O4" to_replaces="O1" bond_order="1"/>
   </residue_link>
   <!-- This link closes the macrocycle -->
   <residue_link from="1" to="6">
     <atom_link from="C1" to="O4" to_replaces="O1" bond_order="1"/>
   </residue_link>
  </molecule>
</GlydeII>
```

3.4.2. Other cyclic structures. Another type of cyclic structure involves the connection of two residues by two distinct bonds, such as the 4,6-acetonide illustrated below.

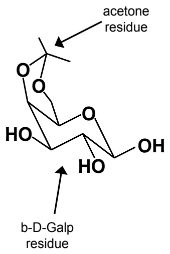


```
<?xml version="1.0" encoding="ISO-8859-1"?>
<!DOCTYPE GlydeII SYSTEM "http://glycomics.ccrc.uga.edu/GLYDE-II/GLYDE-II-1.2.DTD"[
 <!ENTITY mDBget "ref=http://www.monosaccharideDB.org/GLYDE-II.jsp?G">
]>
<GlydeII>
  <molecule id="molecule_1" name="example_acetonide">
   <residue subtype="base_type" partid="1" ref="&mDBget;=b-dgal-hex-1:5"/>
   <residue subtype="substituent" partid="2" ref="&mDBget;=acetonide"
     name="acetonide"/>
   <residue_link from="2" to="1">
     <atom_link from="C2" to="O4" to_replaces="O2a" bond_order="1"/>
     <atom_link from="C2" to="O6" to_replaces="O2b" bond_order="1"/>
   </residue_link>
  </molecule>
</GlydeII>
```

Note that the *residues* in this *molecule* include one **base-type** and one **substituent**. The **substituent** is identified by reference to a molecule called “acetonide”. Since this is a residue of type “substituent” slightly different semantics are used in order to maintain consistency with the GlycoCT namespace. That is, GLYDE-II refers to fully defined molecules as archetypes of residues of subtype “substituent”, while GlycoCT refers to molecular fragments when specifying substituents. GLYDE-II requires an atomistic representation of the substituent, while GlycoCT does not. Therefore, the id of the archetypal substituent molecule is given a name that corresponds to a molecular fragment. In this case, the substituent residue has id=”acetonide”, which has a name corresponding to a molecular fragment but a structure corresponding to an acetone hydrate molecule ($C_3H_6O + H_2O) = C_3H_8O_2$). The link connecting the base-type residue to this substituent *residue* embodies **two** covalent bonds. In this case, “O4” and “O6” of the “b-D-Galp” residue replace “O2a” and “O2b” of the hydrated acetone residue, respectively.

Acetone hydrate is achiral: that is, “O2a” and “O2b” are stereochemically equivalent. However, this is not the case for all acetals of this general type. For example, pyruvate can be linked to a carbohydrate residue via a chiral ketal linkage, as illustrated next.

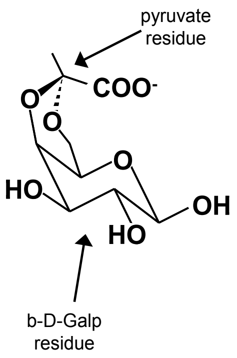

In this case, C2 of the pyruvate is chiral, but this is not the case for free pyruvate. It is necessary to define this substituent-type *residue* by reference to a pyruvate hydrate molecule, in which two different prochiral oxygen atoms, O2R and O2S, are attached to C2. The GLYDE-II representation of this molecule is shown next.

```
<?xml version="1.0" encoding="ISO-8859-1"?>
<!DOCTYPE GlydeII SYSTEM "http://glycomics.ccrc.uga.edu/GLYDE-II/GLYDE-II-1.2.DTD"[
 <!ENTITY mDBget "ref=http://www.monosaccharideDB.org/GLYDE-II.jsp?G">
]>
<GlydeII>
  <molecule id="molecule_1" name="pyruvate ketal">
   <residue subtype="base_type" partid="1" ref="&mDBget;=b-dgal-hex-1:5"/>
   <residue subtype="substituent" partid="2" ref="&mDBget;=pyruvate"
     name="pyruvate"/>
   <residue_link from="2" to="1">
     <atom_link from="C2" to="O4" to_replaces="O2S" bond_order="1"/>
     <atom_link from="C2" to="O6" to_replaces="O2R" bond_order="1"/>
   </residue_link>
  </molecule >
</GlydeII>
```

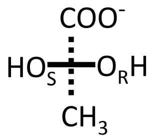

Detail. Prochiral oxygens in the pyruvate molecule

Unless the two oxygens connected to C2 of the pyruvate are stereochemically distinguishable in the molecule containing this substituent *residue*, the pyruvate is achiral. However, in this case, the two oxygen atoms can be distinguished, as the *atom_links* in the above example specify that O4 of the β-D-Galp residue replaces one of the prochiral atoms (O2S) of the pyruvate and O6 of the β-D-Galp residue replaces the other prochiral atom (O2R) of the pyruvate, so the chirality of C2 of the pyruvate *residue* in the context of the *molecule* is fully specified. Thus, pyruvate substituents of this type can have two different stereochemical forms that are distinguished by the values assigned to the "to_replaces" attribute of the *atom_link*.

Extension of **Rule 3** to systematically name oxygen atoms of the hydrated pyruvate archetype *molecule*, unambiguously defines the stereochemistry of this pyruvate ketal. The pro-R oxygen of free pyruvate is so named because replacement of the hydroxyl hydrogen on O2R would result in the R-configuration at C2. (See http://goldbook.iupac.org/P04889.html.) Thus, these two atoms can be distinguished.

3.4.3. Repeating structures. As illustrated in the next example (γ-cyclodextrin), a *residue* can constitute a repeat unit. More generally, a repeating block might contain substructures that are internally linked and the block itself can be linked to other structures. Within the repeat block, one must distinguish the "head-to-tail" link that connects the tandemly-arranged copies of the block to each other from any links that are internal to the block and from links to structures that are external to the block. The *repeat_block* ***element***, which is designed to implement these requirements, contains *repeat_part* ***elements***, each of which embodies a reference to a component of the *repeat_block*. These components are defined (once) outside of the *repeat_block*. The links between these components are also defined outside the *repeat_block*. However, as copies of the the repeat_block are tandemly arranged, the link between each of these tandem repeats must be specified. The head-to-tail link that connects tandem copies of the *repeat_block* is specified within the *repeat_block*. The links connecting the ends of the *repeat_block* to other components of the molecule are specified outside the *repeat_block*. The *repeat_block* has an attribute called *repeat_number*, which indicates how many times the *repeat_block* is tandemly repeated. This is illustrated in the code on the next page.

```
<?xml version="1.0" encoding="ISO-8859-1"?>
<!DOCTYPE GlydeII SYSTEM "http://glycomics.ccrc.uga.edu/GLYDE-II/GLYDE-II-1.2.DTD"[
 <!ENTITY mDBget "http://monosaccharidedb.org/GLYDE-II.jsp?G">
]>
<GlydeII>
 <molecule id="molecule_1" name="gamma_cyclodextrin">
  <residue subtype="base_type" partid="residue_1" ref="&mDBget;=a-dglc-hex-1:5"/>
  <residue subtype="base_type" partid="residue_2" ref="&mDBget;=a-dglc-hex-1:5"/>
  <residue subtype="base_type" partid="residue_3" ref="&mDBget;=a-dglc-hex-1:5"/>
  <!-- the link below specifies a link from a residue
    inside the repeat block TO a residue outside the repeat block -->
  <residue_link from="residue_2" to="residue_1">
   <atom_link from="C1" to="O4" to_replaces="O1" bond_order="1"/>
  </residue_link>

  <!-- the next link specifies a link from a residue
    outside the repeat block TO a residue inside the repeat block -->
  <residue_link from="3" to="2">
   <atom_link from="C1" to="O4" to_replaces="O1" bond_order="1"/>
  </residue_link>

  <!-- the next link specifies a structure that
    is completely outside the repeat block - this closes the macrocycle -->
  <residue_link from="residue_1" to="residue_3">
   <atom_link from="C1" to="O4" to_replaces="O1" bond_order="1"/>
  </residue_link>

  <repeat_block repeat_number_min="6" repeat_number_max="6">
   <residue_ref ref="residue_2"/>
   <!-- head-to-tail link for the end residues of the repeat_block -->
     <!-- the head and tail residues are the same: residue_2 -->
   <residue_link from="residue_2" to="residue_2">
     <atom_link from="C1" to="O4" to_replaces="O1" bond_order="1"/>
   </residue_link>
  </repeat_block>
 </molecule>
</GlydeII>
```

A slightly more complex example is hyaluronan (http://www.chem.qmul.ac.uk/iupac/2carb/39.html#398), in which the repeating block structure is the disaccharide β-D-Glc*p*A-(1-3)-β-D-Glc*p*NAc. The disaccharides are linked together via β-(1-4) linkages from the β-D-GlcpNAc residue of one repeat unit to the β-D-GlcpA of the next. This is illustrated in the code shown on the next page.

```
<?xml version="1.0" encoding="ISO-8859-1"?>
<!DOCTYPE GlydeII SYSTEM "http://glycomics.ccrc.uga.edu/GLYDE-II/GLYDE-II-1.2.DTD"[
 <!ENTITY mDBget "http://www.monosaccharideDB.org/GLYDE-II.jsp?G">
]>
<GlydeII>
  <molecule id="molecule_1" name="hyaluronan">
   <residue subtype="substituent" partid="residue_1" ref="&mDBget;=n-acetyl"/>
   <residue subtype="base_type" partid="residue_2"
      ref="&mDBget;=b-dglc-hex-1:5" name="b-D-Glcp" />
   <residue subtype="base_type" partid="residue_3"
      ref="&mDBget;=b-dglc-hex-1:5,6:a" name="b-D-GlcpA"/>
   <!-- connect the base-type and substituent of the GlcNAc -->
   <residue_link from="residue_1" to="residue_2">
     <atom_link from="N1" to="C2" from_replaces="O2" bond_order="1" />
   </residue_link>
   <!-- the GlcA residue is linked to the GlcNAc residue-->
   <residue_link from="residue_3" to="residue_2">
     <!-- via a 1-3 bond -->
     <atom_link from="C1" to="O3" to_replaces="O1" bond_order="1"/>
   </residue_link>
   <!-- The repeat_block specifies which residues are repeated
      and the link between repeat_blocks
      the maximum number of repeats is not specified -->
   <repeat_block repeat_number_min="2">
     <residue_ref ref="residue_1"/>
     <residue_ref ref="residue_2"/>
     <residue_ref ref="residue_3"/>
     <!-- head-to-tail link for the end residues of the repeat_block -->
     <!-- Specify that GlcN is linked to GlcA to make the tandem repeat -->
     <residue_link from="residue_2" to="residue_3">
      <!-- via a 1-4 bond -->
      <atom_link from="C1" to="O4" to_replaces="O1" bond_order="1"/>
     </residue_link>
   </repeat_block>
  </molecule>
</GlydeII>
```

The hyaluronan polysaccharide consists of tandemly repeated copies of a disaccharide *repeat_unit*, which is specified by listing its *residue* parts and the tandemly repeated residue_link that connects copies of the *repeat_unit*. The disaccharide *repeat_unit* contains three internal *parts* (residues 1, 2 and 3) and two internal *residue_links*. The link between adjacent tandemly repeated copies of the disaccharide is explicitly specified as a link from “C1” of the β-D-GlcpNAc residue in one repeat unit to “O4” of the β-D-GlcpA in the next. Thus, no relationship between the linkages internal to the repeat unit and linkages connecting the tandemly repeated units is assumed and the internal and tandem linkages are fully specified.

3.4.4. Incomplete or statistically known structures. An example of statistically known structural information is chondroitin sulfate, which is related to hyaluronan. Chondroitin sulfate is composed of partially sulfated β-D-GalNAc and β-D-GlcA residues (http://www.ncbi.nlm.nih.gov/books/NBK1900/).

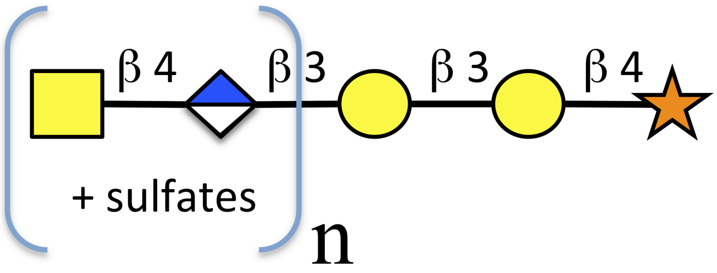

CFG graphical representation of chondroitin sulfate

The sulfate groups are specified as *residue* objects (by reference to the H_2SO_4 archetype molecule, which corresponds to the “so_4” substituent in GlycoCT). The partial presence of sulfate substituents in the chondroitin sulfate repeat unit is specified using an ***attribute*** of the *link* **element** called *stat*, as illustrated in the code on the next page.

```
<?xml version="1.0" encoding="ISO-8859-1"?>
<!DOCTYPE GlydeII SYSTEM " http://glycomics.ccrc.uga.edu/GLYDE-II/GLYDE-II-1.2.DTD"[
 <!ENTITY mDBget "http://www.monosaccharidedb.org/GLYDE-II.jsp?G">
]>
<GlydeII>
  <molecule id="molecule_1" name="chondroitin sulfate">
   <!-- The following parts make up the repeating disaccharide -->
   <residue subtype="substituent" partid="1" ref="&mDBget;=n-acetyl"/>
   <residue subtype="base_type" partid="2" ref="&mDBget;=b-dgal-hex-1:5"/>
   <residue subtype="base_type" partid="3"
       ref="&mDBget;=b-dglc-hex-1:5,6:a" name="b-D-GlcpA"/>
   <residue subtype="substituent" partid="4"
       ref="&mDBget;=sulfuric_acid" name="sulfate"/>
   <residue subtype="substituent" partid="5"
       ref="&mDBget;=sulfuric_acid" name="sulfate"/>

   <!-- The following parts make up the non-repeating core tetrasaccharide -->
   <residue subtype="base_type" partid="6"
      ref="&mDBget;=b-dxyl-pen-1:5" name="b-D-Xylp"/>
   <residue subtype="base_type" partid="7"
      ref="&mDBget;=b-dgal-hex-1:5" name="b-D-Galp"/>
   <residue subtype="base_type" partid="8"
      ref="&mDBget;=b-dgal-hex-1:5" name="b-D-Galp"/>
   <residue subtype="base_type" partid="9"
      ref="&mDBget;=b-dglc-hex-1:5,6:a" name="b-D-GlcpA"/>

   <!-- connect the base-type and substituent of the GalNAc -->
   <residue_link from="1" to="2">
     <atom_link from="N1" to="C2" from_replaces="O2" bond_order="1"/>
   </residue_link>
   <!-- specify that, within the disaccharide, the GlcA is linked to the GalNAc -->
   <residue_link from="3" to="2">
     <!-- via a 1-3 bond -->
     <atom_link from="C1" to="O3" to_replaces="O1" bond_order="1"/>
   </residue_link>
   <!-- 90% of the time, GalNAc's base-type (part 2) has a sulfate (part 4) at O6 -->
   <residue_link from="4" to="2" stat="0.9">
     <atom_link from="S1" to="O6" to_replaces="O1a" bond_order="1"/>
   </residue_link>
   <!-- 5% of the time, GalNAc's base-type (part 2) has a sulfate (part 5) at O4 -->
   <residue_link from="5" to="2" stat="0.05">
     <atom_link from="S1" to="O4" to_replaces="O1a" bond_order="1"/>
   </residue_link>

   <!-- The following links connect the residues within the core tetrasaccharide -->
   <residue_link from="7" to="6">
     <atom_link from="C1" to="O4" to_replaces="O1" bond_order="1"/>
   </residue_link>
   <residue_link from="8" to="7">
     <atom_link from="C1" to="O3" to_replaces="O1" bond_order="1"/>
   </residue_link>
   <residue_link from="9" to="8">
     <atom_link from="C1" to="O3" to_replaces="O1" bond_order="1"/>
   </residue_link>
   <!-- The GalNAc in the disaccharide repeat is linked to GlcA
       in the core tetrasaccharide -->
   <residue_link from="2" to="9">
     <!-- via a 1-4 bond -->
     <atom_link from="C1" to="O4" to_replaces="O1" bond_order="1"/>
   </residue_link>
<!-- CONTINUED ON NEXT PAGE -->
```

```
    <!-- The repeating nature of the disaccharide is specified next -->
    <repeat_block repeat_number="n">
      <repeat_part ref="1"/>
      <repeat_part ref="2"/>
      <repeat_part ref="3"/>
      <repeat_part ref="4"/>
      <repeat_part ref="5"/>
      <!-- head-to-tail for the end residues of the disaccharide -->
      <!-- The tandem repeat is made by linking the GalNAc to GlcA -->
      <residue_link from="2" to="3">
       <!-- via a 1-3 bond -->
       <atom_link from="C1" to="O3" to_replaces="O1" bond_order="1"/>
      </residue_link>
    </repeat_block>
  </molecule>
</GlydeII>
```

This code illustrates several different features of GLYDE-II. Note that two <u>different</u> sulfate residues ("residue_4" and "residue_5") are defined, and the *stat* ***attribute*** of their *links* to the Gal*p*NAc residue specify that the sulfate at O6 is present 90% of the time and the sulfate at O4 is present 5% of the time. (Specifying two different sulfate residues distinguishes this situation from the mutually exclusive case, wherein a single sulfate is present, but it may be in one of two different locations.) This formalism is closely related to that used by Glyco-CT (Herget, Ranzinger et al. 2008) for specifying statistically defined structures of this kind.

Chondroitin sulfate also has a "core tetrasaccharide composed of a β-D-GlcA, two β-D-Gal, and one β-D-Xyl residues. The tandemly repeating, partially sulfated disaccharide is linked to the β-D-GlcA of the core tetrasaccharide.

Sometimes a part is known to be attached to another specific part, but its attachment site is unknown. In this case, the link with coarser granularity is fully specified, but the child link (finer granularity) is specified as a choice. The syntax is similar to that used in Glyco-CT (Herget, Ranzinger et al. 2008) for this situation, using the vertical bar "|", signifying "or". This is illustrated in the code snippet listed below.

```
<residue_link from="residue_6" to="residue_4">
  <atom_link from="C1" to="O6|O2" to_replaces="O1" bond_order="1"/>
</residue_link>
```

This code specifies that the linkage site is partially known (i.e., O6 or O2, but not O3). In general, what is implicitly known should be specified. If it is known, for example, that the attachment is by an O-glycosidic linkage *to* an aldohexopyranose, then one should specify `to="O2|O3|O4|O6"` (i.e., it cannot be to O5 of the pyranose.) As in Glyco-CT, the "?" is not allowed for the values of the *to* and *from* ***attributes*** of a *link*.

There exists another type of uncertainty, where a *residue* or group of *residues* are known to be present, but their precise location is unknown. This type of uncertainty is indicated by the "PRO section" of Glyco-CT. The GLYDE-II formalism is similar but uses an ***element*** called *combination*. Consider, for example, a N-linked glycan that contains a single terminal β-D-GlcpNAc *residue*, which may be attached at any one of the four following sites: (i) O6 of the α-D-Manp on the 6-arm, (ii) O2 of the α-D-Manp on the 6-linked arm, (iii) O4 of the α-D-Manp on the 3-linked arm, and (iv) O2 of the α-D-Manp

on the 3-linked arm. Thus, both the residue-level site and the atomic-level site are unknown, but not completely unknown. That is, it is known that the β-D-GlcpNAc *residue* (e.g, with `partid="6"`) is attached to O6 or O2 of one α-D-Manp residue (e.g, with `partid="4"`) or O4 or O2 of another α-D-Manp residue (e.g, with `partid="5"`). This uncertainty is represented by the following code snippet.

```
<combination>
  <residue_link from="6" to="4">
   <atom_link from="C1" to="O6|O2" to_replaces="O1" bond_order="1"/>
  </residue_link>
  <residue_link from="6" to="5">
   <atom_link from="C1" to="O4|O2" to_replaces="O1" bond_order="1"/>
  </residue_link>
</combination>
```

The ***element*** called *combination* is a collection of different links that represent all of the possible partially known attachment sites. In this example, the *combination* specifies the uncertainty of locating a single part (i.e., the β-D-GlcpNAc *residue* with `partid="6"`) that can be attached by any of the linkages enclosed in the tag. This *combination* must contain two different *residue_links*, as the *residue_link* to residue_4 is via O6 or O2, while the *residue_link* to residue_5 is via O4 or O2. Since this *combination* has only one *part*, only one of the possible linkage sites can be occupied. As with the "PRO section" of the Glyco-CT formalism, a combination can only be used to represent homogenic structures (where only one structure exists, but its precise structure is unknown). Mixtures should be represented by a collection of crisp representations.

Alternatively, two β-D-GlcpNAc residues (one with `partid="6"` and one with `partid="7"`) may be present at the same set of four possible sites described above, but their precise locations are not known. Obviously, they cannot both be present at the same location, and this is where the *combination* ***element*** is most useful. This is illustrated in the code snippet below.

```
<!-- this specifies the possible sites for residue 6 and residue 7 -->
<!-- Only 1 combination is true -->
<combination>
  <residue_link from="6" to="4">
   <atom_link from="C1" to="O6|O2" to_replaces="O1" bond_order="1"/>
  </residue_link>
  <residue_link from="6" to="5">
   <atom_link from="C1" to="O4|O2" to_replaces="O1" bond_order="1"/>
  </residue_link>
  <residue_link from="7" to="4">
   <atom_link from="C1" to="O6|O2" to_replaces="O1" bond_order="1"/>
  </residue_link>
  <residue_link from="7" to="5">
   <atom_link from="C1" to="O4|O2" to_replaces="O1" bond_order="1"/>
  </residue_link>
</combination>
```

This is the case that is most similar to the case used to describe the "PRO section" of Glyco-CT. A parsing algorithm could easily enumerate all of the possible combinations implicit in this representation, excluding those where the same site is occupied by two different structures.

This formalism would also be very useful in describing the structure of fragment ions observed in MS/MS spectra. For example, a fully methylated oligosaccharide ion is fragmented and it is known that a particular fragment ion contains an α-D-Manp residue that has a β-D-GlcpNAc *residue* attached at O2, O3, O4, or O6 and methyl substituents (which are formally *residues*) attached to the oxygens that do not bear the β-D-GlcpNAc residue. That is, the attachment site of the β-D-GlcpNAc residue in the original structure of the parent ion is not known, but the attachment sites of the β-D-GlcpNAc and methyl residues are mutually exclusive. Then, a *combination* whose parts are 3 methyl residues and the β-D-GlcpNAc residue can be specified, and this combination would contain the possible (mutually exclusive) links to the α-D-Manp residue. The code would look like the following:

```
<residue subtype=”substituent” partid="3" ref="&mDBget;=n-acetyl"/>
<residue subtype=”base_type” id="4" ref="&mDBget;=b-dman-hex-1:5"/>
<residue subtype=”base_type” id="5" ref="&mDBget;=b-dglc-hex-1:5"/>
<residue subtype=”substituent” id="6" ref="&mDBget;=methyl"/>
<residue subtype=”substituent” id="7" ref="&mDBget;=methyl "/>
<residue subtype=”substituent” id="8" ref="&mDBget;=methyl "/>
...........
<combination parts="5|6|7|8>
  <!-- this specifies the possible sites for residues 5-8 -->
  <!-- only one combination is true -->
  <link from="5|6|7|8" to="4">
   <link from="C1" to="O2|O3|O4|O6" to_replaces="O1" bond_order="1"/>
  </link>
</combination>
```

In this case, the methyl groups are based on a methanol *molecule* and O1 of the methanol is replaced by O2 or O3 or O4 or O6 of the α-D-Manp residue when the *links* are instantiated. One disadvantage of this approach is that, for combinations that include chemically identical structures, several degenerate combinations of mutually exclusive links are possible. That is, the combination in which (methyl) residue 6 is at O2 and (methyl) residue 7 is at O3 is formally distinct from the converse combination in which (methyl) residue 6 is at O3 and (methyl) residue 7 is at O2, even though these two combinations actually have the same chemical structure. This degeneracy is also a characteristic for the above example with two different β-D-GlcpNAc residues. It also appears to be a characteristic of Glyco-CT when ambiguity is represented using the “PRO section”.

One might imagine that collecting these links within a *combination* ***element*** is unnecessary. However, it is possible that a single molecule can have more than one set of structures with mutually exclusive attachment sites, and these sets must be logically separated by specifying more than one *combination*.

4. Molecular geometry

A basic discussion of the GLYDE-II representation of molecular geometry is presented in the main text of the manuscript describing GLYDE-II, including both explicit (Cartesian) representations invoking the *x*, *y* and *z* ***attributes*** of the *coords* tag and abstract

representations invoking the *id* ***attribute*** of a GLYDE *molecule*. Often, the geometric information included in the *id* ***attribute*** of a *molecule* is sufficient to make valuable inferences about the molecular structure and its relationships to its biological and physical properties. However, explicit representations of molecular geometry can be used to define geometry at the atomic level when this is appropriate. That is, conventions are specified to facilitate the implementation of algorithms to interconvert GLYDE representations and fully atomistic representations. The coordinates of each atom in the global frame of reference can be inferred from parameters that translate and rotate the molecule from the local frame of reference to the global frame of reference. A global frame of reference is most easily defined by specifying an *aggregate* to hold a *molecule_instance* so that the molecule's *x*-, *y*- and *z*-coordinates in the aggregate's frame of reference can be assigned. For example, the local coordinates of each atom in an α-D-Manp molecule are defined in its GLYDE representation. A *molecule_instance* defined by reference to the α-D-Manp *molecule* can be placed at any Cartesian coordinates in a global frame of reference that is arbitrarily defined by the *aggregate*. However, in order to infer the global coordinates of all of the atoms in the α-D-Manp *molecule_instance*, its orientation must also be specified. This is accomplished by specifying a set of three Euler angles that rotate the *molecule_instance* in the global frame of reference.

A

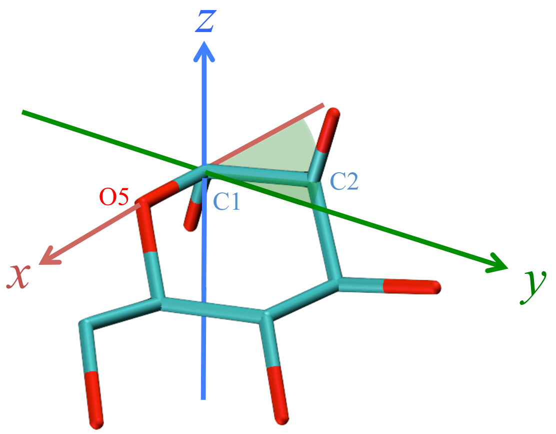

B

```
1 <GlydeII>
2  <molecule subtype="base_type" id="a-dman-hex-1:5" name="a-D-Manp">
3   <bound_atom partid="C1"
             ref=" http://glycomics.ccrc.uga.edu/GLYDE-II/lib/atoms.xml#C">
4    <coords x="0.000" y="0.000" z="0.000" />
5   </bound_atom>
6   <bound_atom partid="C2"
             ref="http://glycomics.ccrc.uga.edu/GLYDE-II/lib/atoms.xml#C">
7    <coords x="-0.509" y="1.444" z="0.000" />
8   </bound_atom>
     ...
33  <bound_atom partid="O5" ref="#O"
             ref="http://glycomics.ccrc.uga.edu/GLYDE-II/lib/atoms.xml#O">
34   <coords x="1.428" y="0.000" z="0.000" />
35  </bound_atom>
     ...
60  <atom_link from="C2" to="C1" bond_order="1" />
     ...
69  <atom_link from="O5" to="C1" bond_order="1" />
     ...
79 </molecule>
80 </GlydeII>
```

Detail. Stick model (**A**) and abbreviated GLYDE-II representation (**B**) of an α-D-Man*p* *molecule*. (Several lines of XML code are omitted for brevity; the remaining lines are numbered.) The structure of each *bound_atom* is specified by its *ref* attribute (lines 3, 6 and 33), which points to a GLYDE-II representation of the *atom* serving as the archetype for the *bound_atom*. The molecular topology is fully specified by *atom_link* objects (e.g., lines 60 and 69), which connect *bound_atom* objects. The molecular configuration

(stereochemistry) is specified explicitly by listing the coordinates of each *bound_atom*. Alternatively, stereochemistry of the *molecule* can be inferred from its *id* (line 2), which by rule corresponds to its representation using a format based on GlycoCT. The conventional orientation and position of the Cartesian axes in the atomistic GLYDE-II representation is defined by the alignment of three key *bound_atom* objects: the anomeric carbon (C1 in this case) is at the origin, the ring oxygen (O5 in this case) is on the *x*-axis, and the highest-numbered carbon (C2 in this case) that is directly linked to the anomeric carbon is in the first or second quadrant of the *x,y*-plain (where $y > 0$).

Given the global coordinates of the atoms in a molecule (*e.g.*, as a pdb file) and a method to identify the partonomic relationships in that molecule, it is a straightforward task to generate a GLYDE representation of the molecule in which each part is properly located and aligned within its local (*aggregate, molecule, moiety, residue*) frame of reference. This recursive process (starting with the coarsest granularity) involves a translation of each part to establish its local Cartesian origin followed by a rotation to properly orient it in its local Cartesian frame of reference. The conventional orientation of the *bound_atoms* in a *molecule* is specified in the above Figure. The conventional orientation of the *residues* in a *moiety* is defined below. Once the translation parameters (Δx, Δy and Δz) and rotation matrix are calculated for this process, it is straightforward to calculate (at each level of granularity) the local coordinates (*x*, *y* and *z*) and Euler angles (α, β and γ) required to generate a parameterized GLYDE representation that can be used to reproduce the fully atomistic representation. In this context, it is important to note that, as defined in GLYDE, each *bound_atom* and *free_atom* is a so-called “structureless particle”, so its orientation in the molecule’s local frame of reference is undefined. Thus, *bound_atom* and *free_atom_instance* objects have no attributes corresponding to Euler rotation angles.

Translation of a GLYDE representation of a complex molecule into a fully atomistic representation is also a recursive process that, in this case, involves rotation of each part (as specified by the part’s Euler angles) followed by translation (as specified by the part’s Cartesian coordinates). For example, given an *aggregate* consisting of a single *molecule_instance* specified by reference to a *molecule* composed of *bound_atoms*, the *aggregate* (global) coordinates of each *bound_atom* in the aggregate are readily calculated. One starts with the local coordinates of each *bound_atom* in the *molecule* that was used as an archetype for the *molecule_instance*. The *bound_atoms* are rotated about the local origin using the Euler angle attributes (alpha, beta and gamma) of the *molecule_instance*, and then translated such that that the atom at the local origin (e.g., the anomeric carbon of the archetype *molecule*) is translated in the global frame to the coordinates specified by the *x, y,* and *z* attributes of the *molecule_instance*. The identical algorithm can be used, given the local coordinates of each *bound_atom* in a *residue* of an enclosing *moiety*, and the coordinates and Euler angles of the *residue* in the *moiety*, to calculate the coordinates of each of the residue’s atoms in the *moiety* frame of reference. With that information, along with the coordinates and Euler angles of the *moiety* in the context of the *molecule*, the same algorithm can be used to calculate the coordinates of the atoms in the frame of reference of the *molecule*. The algorithm can be applied again if

the *molecule* (containing the *moiety*) is used as an archetype for an oriented *molecule_instance* in an *aggregate*, as described at the top of this paragraph. This recursive procedure is a good example of how the hierarchical self-consistency of structural representations in GLYDE allows structural information to be processed at different levels of granularity using the same algorithm.

Conventions for Rotation. The expressions shown here are consistent with those presented by Arfken (Arfken, Weber et al. 2012) and with Weisstein at Wolfram Research (http://mathworld.wolfram.com/EulerAngles.html. (See also http://mathworld.wolfram.com/RotationMatrix.html.) Weisstein also gives a recipe for determining the Euler angles for transforming an object in one coordinate system to another coordinate system (by rotation) given coordinates of several points in the object in both systems.

Rotation of **axes** by an angle ϕ:

$$\mathbf{R}_x(\phi) = \begin{pmatrix} 1 & 0 & 0 \\ 0 & \cos\phi & \sin\phi \\ 0 & -\sin\phi & \cos\phi \end{pmatrix} \quad \mathbf{R}_y(\phi) = \begin{pmatrix} \cos\phi & 0 & -\sin\phi \\ 0 & 1 & 0 \\ \sin\phi & 0 & \cos\phi \end{pmatrix} \quad \mathbf{R}_z(\phi) = \begin{pmatrix} \cos\phi & \sin\phi & 0 \\ -\sin\phi & \cos\phi & 0 \\ 0 & 0 & 1 \end{pmatrix}$$

A positive value of ϕ corresponds to a counterclockwise rotation of the **axes** when viewed from the direction of the (positive) axis of rotation. This is referred to a right-hand “alias” convention, as vectors are stationary but given different names when the axes are rotated. Rotation of the vectors themselves is called “alibi” rotation, as the vectors themselves move (they are somewhere else.)

For rotation to obtain an arbitrary orientation, three Euler angles α, β, and γ, can be defined. In the following, c_1 corresponds to $\cos(\alpha)$, s_2 corresponds to $\sin(\beta)$, etc.

The order in which the rotations are applied is opposite the order they are written. Thus, for the *zxz* (alias) convention (Arfken, Weber et al. 2012), the following hold true

$$\mathbf{R}_z(\alpha) = \begin{pmatrix} c_1 & s_1 & 0 \\ -s_1 & c_1 & 0 \\ 0 & 0 & 1 \end{pmatrix} \qquad \mathbf{R}_x(\beta) = \begin{pmatrix} 1 & 0 & 0 \\ 0 & c_2 & s_2 \\ 0 & -s_2 & c_2 \end{pmatrix} \qquad \mathbf{R}_z(\gamma) = \begin{pmatrix} c_3 & s_3 & 0 \\ -s_3 & c_3 & 0 \\ 0 & 0 & 1 \end{pmatrix}$$

$$\mathbf{R} = \mathbf{R}_z(\gamma)\mathbf{R}_x(\beta)\mathbf{R}_z(\alpha) = \begin{pmatrix} c_3 & s_3 & 0 \\ -s_3 & c_3 & 0 \\ 0 & 0 & 1 \end{pmatrix} \begin{pmatrix} 1 & 0 & 0 \\ 0 & c_2 & s_2 \\ 0 & -s_2 & c_2 \end{pmatrix} \begin{pmatrix} c_1 & s_1 & 0 \\ -s_1 & c_1 & 0 \\ 0 & 0 & 1 \end{pmatrix}$$

$$\mathbf{R} = \begin{pmatrix} c_3 & s_3 & 0 \\ -s_3 & c_3 & 0 \\ 0 & 0 & 1 \end{pmatrix} \begin{pmatrix} c_1 & s_1 & 0 \\ -s_1 c_2 & c_1 c_2 & s_2 \\ s_1 s_2 & -c_1 s_2 & c_2 \end{pmatrix} = \begin{pmatrix} c_1 c_3 - s_1 c_2 s_3 & s_1 c_3 + c_1 c_2 s_3 & s_2 s_3 \\ -c_1 s_3 - s_1 c_2 c_3 & -s_1 s_3 + c_1 c_2 c_3 & s_2 c_3 \\ s_1 s_2 & -c_1 s_2 & c_2 \end{pmatrix}$$

Right-multiplication of the (alias) matrix $\mathbf{R} = \mathbf{R}_z(\gamma)\mathbf{R}_x(\beta)\mathbf{R}_z(\alpha)$ by the column vector gives the transformed coordinates obtained by rotating the axes. For example, for the point (1,1,0) [expressed as a column vector] using $\alpha = \pi/2$, $\beta = \pi/2$ and $\gamma = 0$, and the *zxz* alias convention,

$$\mathbf{R} = \mathbf{R}_z(\gamma)\mathbf{R}_x(\beta)\mathbf{R}_z(\alpha) = \begin{pmatrix} 0 & 1 & 0 \\ 0 & 0 & 1 \\ 1 & 0 & 0 \end{pmatrix}$$

Right-multiplying this by the coordinate vector gives

$$\mathbf{R}v = \begin{pmatrix} 0 & 1 & 0 \\ 0 & 0 & 1 \\ 1 & 0 & 0 \end{pmatrix} \begin{pmatrix} 1 \\ 1 \\ 0 \end{pmatrix} = \begin{pmatrix} 1 \\ 0 \\ 1 \end{pmatrix}$$

This agrees with the manually generated results for the new coordinates when performing the rotation of the **axes** using these Euler angles, as shown below.

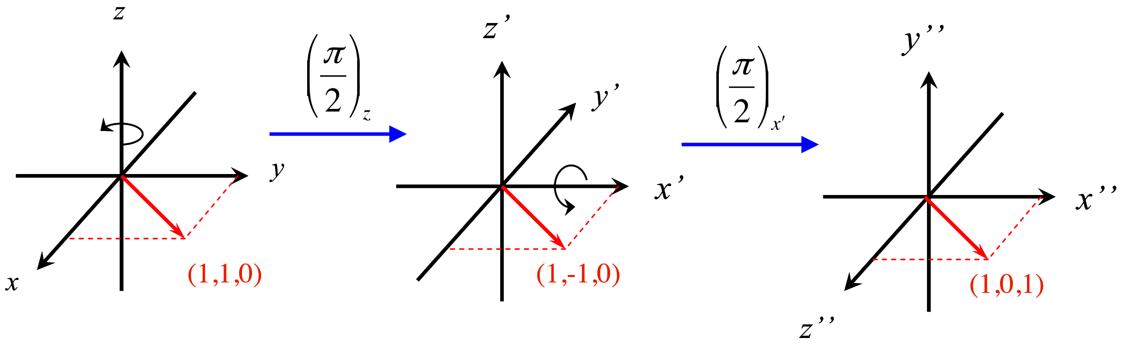

Rotating vectors is the same as rotating axes, **but in the opposite direction**. That is, when the alias convention is used, a positive value of ϕ corresponds to a clockwise rotation of the **vector** when viewed from the direction of the (positive) axis of rotation.

For example, for the same point (1,1,0) [expressed as a column vector] using the same angles $\alpha = \pi/2$, $\beta = \pi/2$ and $\gamma = 0$, and the *zxz* alias convention, the same rotation matrix is obtained.

$$\mathbf{R} = \mathbf{R}_z(\gamma)\mathbf{R}_x(\beta)\mathbf{R}_z(\alpha) = \begin{pmatrix} 0 & 1 & 0 \\ 0 & 0 & 1 \\ 1 & 0 & 0 \end{pmatrix}$$

Right-multiplying this by the coordinate vector gives the same answer as before:

$$\mathbf{R}v = \begin{pmatrix} 0 & 1 & 0 \\ 0 & 0 & 1 \\ 1 & 0 & 0 \end{pmatrix}\begin{pmatrix} 1 \\ 1 \\ 0 \end{pmatrix} = \begin{pmatrix} 1 \\ 0 \\ 1 \end{pmatrix}$$

This agrees with the manually derived answer for the new coordinates when performing the rotation of the **vector** using these Euler angles, as shown below.

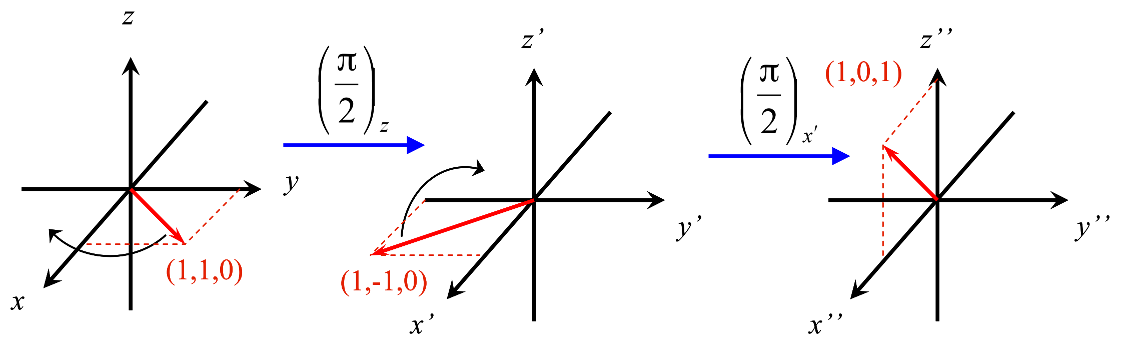

In conclusion, for rotation of axes or vectors using the alias convention, the same Euler rotation matrix is right-multiplied by the column vector describing the coordinates of the point. **According to the original rotation matrix definitions above [i.e., $R_x(\phi)$, $R_y(\phi)$, and $R_z(\phi)$], a positive angle ϕ corresponds to counterclockwise rotation of an axis and to the clockwise rotation of a vector, looking toward the origin from the (positive) rotation axis**.

Alternatively, one could redefine the rotation matrices for vectors, as done by Weisstein. (see http://mathworld.wolfram.com/RotationMatrix.html.) In this case, the rotation is said to be an "alibi" (the vector is somewhere else), and the rotation matrix for a vector about a particular axis would be the transpose of the alias rotation matrix about that axis. (This can be derived from the fact that rotation matrices are orthogonal.) The relevant rotation matrices are given below.

(Alibi) rotation of **vectors** by an angle ϕ:

$$\mathbf{R}_x(\phi) = \begin{pmatrix} 1 & 0 & 0 \\ 0 & \cos\phi & -\sin\phi \\ 0 & \sin\phi & \cos\phi \end{pmatrix} \quad \mathbf{R}_y(\phi) = \begin{pmatrix} \cos\phi & 0 & \sin\phi \\ 0 & 1 & 0 \\ -\sin\phi & 0 & \cos\phi \end{pmatrix} \quad \mathbf{R}_z(\phi) = \begin{pmatrix} \cos\phi & -\sin\phi & 0 \\ \sin\phi & \cos\phi & 0 \\ 0 & 0 & 1 \end{pmatrix}$$

For alibi rotations, a positive angle corresponds to a counterclockwise rotation of the **vector** when looking toward the origin from the positive rotation axis.

For the *zxz* convention using the alibi rotation definitions, the following hold true

$$\mathbf{R}_z(\alpha) = \begin{pmatrix} c_1 & -s_1 & 0 \\ s_1 & c_1 & 0 \\ 0 & 0 & 1 \end{pmatrix} \qquad \mathbf{R}_x(\beta) = \begin{pmatrix} 1 & 0 & 0 \\ 0 & c_2 & -s_2 \\ 0 & s_2 & c_2 \end{pmatrix} \qquad \mathbf{R}_z(\gamma) = \begin{pmatrix} c_3 & -s_3 & 0 \\ s_3 & c_3 & 0 \\ 0 & 0 & 1 \end{pmatrix}$$

$$\mathbf{R} = \mathbf{R}_z(\gamma)\mathbf{R}_x(\beta)\mathbf{R}_z(\alpha) = \begin{pmatrix} c_3 & -s_3 & 0 \\ s_3 & c_3 & 0 \\ 0 & 0 & 1 \end{pmatrix} \begin{pmatrix} 1 & 0 & 0 \\ 0 & c_2 & -s_2 \\ 0 & s_2 & c_2 \end{pmatrix} \begin{pmatrix} c_1 & -s_1 & 0 \\ s_1 & c_1 & 0 \\ 0 & 0 & 1 \end{pmatrix}$$

$$\mathbf{R} = \begin{pmatrix} c_3 & -s_3 & 0 \\ s_3 & c_3 & 0 \\ 0 & 0 & 1 \end{pmatrix} \begin{pmatrix} c_1 & -s_1 & 0 \\ s_1c_2 & c_1c_2 & -s_2 \\ s_1s_2 & c_1s_2 & c_2 \end{pmatrix} = \begin{pmatrix} c_1c_3 - s_1c_2s_3 & -s_1c_3 - c_1c_2s_3 & s_2s_3 \\ c_1s_3 + s_1c_2c_3 & -s_1s_3 + c_1c_2c_3 & -s_2c_3 \\ s_1s_2 & c_1s_2 & c_2 \end{pmatrix}$$

Such alibi rotations are more appropriate for rotation of real objects like molecules and their parts.

For example, for the same point (1,1,0) [expressed as a column vector] using the same angles $\alpha = \pi/2$, $\beta = \pi/2$ and $\gamma = 0$, and the *zxz* (alibi) convention, the following rotation matrix is obtained.

$$\mathbf{R} = \mathbf{R}_z(\gamma)\mathbf{R}_x(\beta)\mathbf{R}_z(\alpha) = \begin{pmatrix} 0 & -1 & 0 \\ 0 & 0 & -1 \\ 1 & 0 & 0 \end{pmatrix}$$

Right-multiplying this alibi rotation matrix by the coordinate vector gives the following result:

$$\mathbf{R}v = \begin{pmatrix} 0 & -1 & 0 \\ 0 & 0 & -1 \\ 1 & 0 & 0 \end{pmatrix} \begin{pmatrix} 1 \\ 1 \\ 0 \end{pmatrix} = \begin{pmatrix} -1 \\ 0 \\ 1 \end{pmatrix}$$

This agrees with the manually derived result for the new coordinates when performing the alibi rotation of the **vector** using these Euler angles, as shown below.

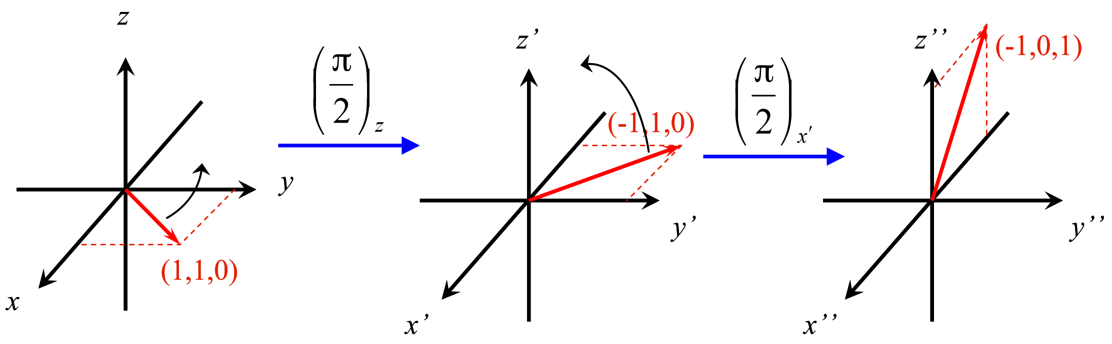

Conclusion: The zxz alibi convention corresponds to the counterclockwise rotation of vectors when looking toward the origin from the positive rotation axis. This corresponds to a clockwise rotation when viewed from the origin, looking toward the positive rotation axis. This is the most natural convention, as it is similar to the physical convention (http://hyperphysics.phy-astr.gsu.edu/hbase/rotv.html) for defining angular velocity. **The zxz alibi convention was thus selected as the convention for rotation of objects in GLYDE.**

References

Arfken, G. B., H. J. Weber and F. E. Harris (2012). Mathematical Methods for Physicists: A Comprehensive Guide, Elsevier.
Casati, R. and A. C. Varzi (1999). Parts and Places: The Structures of Spatial Representations. MIT Press.
Herget, S., R. Ranzinger, K. Maass and C.-W. von der Lieth (2008). "GlycoCT-a unifying sequence format for carbohydrates." Carbohydrate Research **343**, 2162–2171. doi:10.1016/j.carres.2008.03.011
